# Sharp Tuning of Head Direction and Angular Head Velocity Cells in the Somatosensory Cortex

**DOI:** 10.1002/advs.202200020

**Published:** 2022-03-17

**Authors:** Xiaoyang Long, Bin Deng, Calvin K. Young, Guo‐Long Liu, Zeqi Zhong, Qian Chen, Hui Yang, Sheng‐Qing Lv, Zhe Sage Chen, Sheng‐Jia Zhang

**Affiliations:** ^1^ Department of Neurosurgery Xinqiao Hospital Army Medical University Chongqing 400037 China; ^2^ Department of Psychology Brain Health Research Centre University of Otago Dunedin 9054 New Zealand; ^3^ Center for Biomedical Analysis College of Basic Medicine Army Medical University Chongqing 400038 China; ^4^ Department of Psychiatry Department of Neuroscience and Physiology Neuroscience Institute New York University School of Medicine New York NY 10016 USA

**Keywords:** angular head velocity cells, fast‐spiking neurons, head direction cells, somatosensory cortex, spatial cognitive maps, theta rhythmicity

## Abstract

Head direction (HD) cells form a fundamental component in the brain's spatial navigation system and are intricately linked to spatial memory and cognition. Although HD cells have been shown to act as an internal neuronal compass in various cortical and subcortical regions, the neural substrate of HD cells is incompletely understood. It is reported that HD cells in the somatosensory cortex comprise regular‐spiking (RS, putative excitatory) and fast‐spiking (FS, putative inhibitory) neurons. Surprisingly, somatosensory FS HD cells fire in bursts and display much sharper head‐directionality than RS HD cells. These FS HD cells are nonconjunctive, rarely theta rhythmic, sparsely connected and enriched in layer 5. Moreover, sharply tuned FS HD cells, in contrast with RS HD cells, maintain stable tuning in darkness; FS HD cells’ coexistence with RS HD cells and angular head velocity (AHV) cells in a layer‐specific fashion through the somatosensory cortex presents a previously unreported configuration of spatial representation in the neocortex. Together, these findings challenge the notion that FS interneurons are weakly tuned to sensory stimuli, and offer a local circuit organization relevant to the generation and transmission of HD signaling in the brain.

## Graphical abstract

Head direction cells act as an internal neuronal compass in the brain's spatial navigation system. However, the neuronal substrate of head direction cells remains poorly understood. Long and co‐workers first identify sharply tuned fast‐spiking head direction cells in somatosensory cortex that fire in bursts. Their findings uncover the cellular basis for somatosensory head direction cells different from their classical hippocampal counterpart.

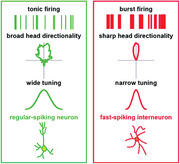

## 1 Introduction

The ability to navigate from one place to another requires the knowledge of one's location and orientation in space. While the hippocampal‐entorhinal system appears to map out the spatial environment,^[^ ^1^, ^2^, ^3^ ^]^ the head direction (HD) system maintains an internally generated reference point to anchor our orientation in space. Spiking activities associated with HD were first reported in the rat postsubiculum^[^ ^4^ ^]^ and were strongly modulated by salient visual cues.^[^ ^4^, ^5^ ^]^ The neural substrate of HD cell activity is dependent on vestibular input.^[^ ^6^, ^7^ ^]^ The existence of cells responsive to the speed of angular head displacement (angular head velocity; AHV cells) and connection patterns between the lateral mammillary nucleus and the dorsal tegmental nucleus prompted the suggestion that a ring attractor network involving these structures may support generation of HD signaling throughout the brain.^[^ ^8^, ^9^ ^]^

The importance of the HD system in the spatial representation of the brain is highlighted by the persistence of HD representation upon manipulations that disrupt periodic firing of entorhinal grid cells.^[^ ^10^, ^11^ ^]^ HD representations survive in cells that encode HD conjunctively with grid representations.^[^ ^10^ ^]^ Conversely, the elimination of HD signal into parahippocampal cortices through anterodorsal thalamic nucleus lesions significantly disrupts HD, place^[^ ^6^, ^12^, ^13^ ^]^ and grid^[^ ^14^ ^]^ cell signals. Therefore, available evidence suggests that HD signaling goes beyond egocentric spatial representation and is also crucial for maintaining allocentric spatial representation.

It is known that HD cells cannot maintain stable tuning once animals are deprived of sensorimotor input through passive transport by a cart.^[^ ^15^ ^]^ Further, passive rotation of the animal led to decreased firing rates of HD cells, despite the preservation of directional tuning^[^ ^5^, ^16^, ^17^ ^]^ attributable to a mismatch of sensorimotor signals from insufficient head restraint.^[^ ^18^ ^]^ These studies suggest that self‐motion might be important for sharpening head directional tuning.^[^ ^19^ ^]^ Similar tuning properties also exist for AHV cells, where passive rotation abolishes AHV tuning in a subset of cells.^[^ ^20^ ^]^ Collectively, these data point to the importance of sensorimotor input in the maintenance and persistence of HD and AHV activity.

Spatial selectivity in the brain was previously assumed to be a property of specialized regions centering around the temporal cortex. More recent investigations have suggested spatial representation may be more widespread than previously thought.^[^ ^21^, ^22^, ^23^ ^]^ We discovered that many spatial cell types can be found in the rat primary somatosensory cortex (S1), including HD cells.^[^ ^23^ ^]^ In the current study, we found that somatosensory HD cells could be classified into regular‐spiking (RS), putative excitatory and fast‐spiking (FS), putative inhibitory neurons. Remarkably, HD representation is not exclusive to RS putative principal cells in the S1, and FS putative interneurons are proportionally over‐represented in HD‐tuned cells from the S1. In addition, FS HD cells have sharper HD tuning, appear to be enriched in layer 5 of the S1, and exhibit sparsely local functional connectivity. AHV tuning is also evident in both FS and RS populations, with proportionally more FS cells displaying AHV tuning than RS counterparts. Moreover, FS cells show better AHV tuning than RS cells, and are recorded across layers 4–6 in the S1. Our findings indicate inhibitory interneurons can be better tuned to sensory inputs than principle cells, in contrast to previous reports that inhibitory interneurons in the cortex had weak and broad tuning to sensory inputs.^[^ ^24^, ^25^ ^]^ These finely tuned FS HD and FS AHV signals in the S1, in combination with their local functional connectivity and patterned distribution across cortical layers, pose a novel local circuit configuration for the internal representation of space.

## 2 Results

### 2.1 Somatosensory Regular‐Spiking and Fast‐Spiking HD cells

**Figure 1:**
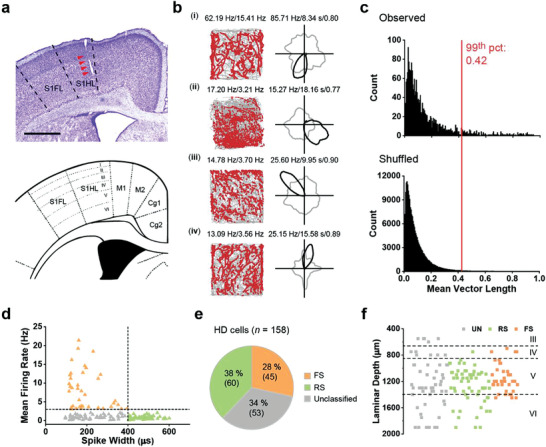
Both regular‐spiking (RS) and fast‐spiking (FS) cells are tuned to head direction in the S1. a) A representative Nissl‐stained coronal section shows tetrode track (indicated with red arrowheads) through the rat S1 (top). Idealized S1 layer boundaries are depicted on the reference atlas (bottom). Scale bar, 1 mm. b) Four representative somatosensory HD cells. Trajectory (grey line) with superimposed spike locations (red dots; left), and HD tuning curves (black) plotted against dwell‐time (gray) in polar coordinates (right). Peak firing rate (fr), mean fr, peak angular fr, peak dwell time, and mean vector length for each representative HD cell are labeled at the top of the plots. The peak dwell time is marked in seconds. c) Distribution of mean vector length for the entire pool of recorded somatosensory cells. The top panel shows the distribution for observed values. The bottom panel shows the distribution for randomly shuffled data from the same pool of recorded S1 cells. d) Distribution of the mean firing rate versus peak‐to‐trough duration (spike width) delineates three types of HD cells: regular‐spiking (RS; green), fast‐spiking (FS; orange) and the unclassified (UN; grey) cells. e) Pie chart showing the proportion of three types of HD cells. f) Reconstructed recording depth for all S1 HD cells. Dashed lines delineate putative layer borders.

Putative single‐cell recordings (Figure S1a,b, Supporting Information) through the S1 (**Figure** **1**a and Figure S2, Supporting Information) were obtained from eleven rats performing a pellet chasing task in an open field (1 m × 1 m). Spike sorting and isolation quality were quantified by calculating the *L*‐ratio and the isolation distance^[^ ^26^ ^]^ for all recorded units (Figure S1c,d, Supporting Information). To ensure that the recorded units were well‐isolated from other spikes simultaneously recorded on the same tetrode, we only considered 2112 units that met our unit isolation threshold with *L*‐ratio < 1.

As described previously,^[^ ^23^ ^]^ HD tuned cells can be found in the S1 (Figure 1b). Neurons with mean vector length exceeding the 99^th^ percentile of the shuffled population distribution (0.42) were classified as HD cells (Figure 1c). Using the above criteria, we reported that 158/2112 (7.5%) of the cells were classified as HD cells. The HD selectivity was stable within each recording session (Figure S3, Supporting Information) and was not biased by rats’ location in the arena (Figure S4, Supporting Information). To account for the possible bias of non‐uniform distribution of dwell time on the directional firing of HD cells, we further calculated the mean vector length of dwell time for HD cells in the S1 (Figure S5c, Supporting Information). The mean vector length of dwell time was significantly lower than that of HD tuning, indicating a uniform distribution of dwell time.

To examine the relative contribution of putative excitatory and inhibitory neurons, we classified the recorded neurons based on their spike widths and firing rates, and further designated spike clusters into regular spiking (RS; putative excitatory), fast‐spiking (FS; putative inhibitory), and unclassified (UN) neurons (Figure 1d and Figure S6a,b, Supporting Information). Specifically, 525/2112 (25%) of recorded cells were classified as FS putative interneurons. Previous studies reported that FS interneurons constituted 12%–25% of the total neuron population in the rat somatosensory cortex, with the percentage varying substantially between cortical layers and reaching the highest percentage in layers 2 and 5A.^[^ ^27^, ^28^, ^29^ ^]^ The relatively high percentage of FS cells in our study could be a combination of two reasons: 1) electrodes mainly targeted to deep layers (L4‐6); 2) sampling biases of extracellular recordings such as the tendency to record from more active FS cells.^[^ ^30^ ^]^

Among 158 identified HD cells, 60/158 (38.0%) were RS HD cells, 45/158 (28.5%) were FS HD cells, and 53/158 (33.5%) were UN HD cells (Figure 1e). The number of each type of HD cells recorded across animals was summarized in Table S1, Supporting Information. Among all classified FS, RS, and UN cells, we found 60/840 (7.1%) of RS cells, 45/525 (8.6%) of FS cells, and 53/747 (7.1%) of UN cells that displayed HD tuning. The proportions of HD cells among RS, FS and UN cells were significantly larger than expected by chance (RS, *Z* = 17.9, *P* < 0.001; UN, *Z* = 16.7, *P* < 0.001; FS, *Z* = 17.4, *P* < 0.001; binomial tests with expected *P*_0_ of 0.01).

We found 52% of the RS HD cells with no detectable conjunctive representation, but none of the FS HD cells showed detectable conjunctive representation (Figure S5a, Supporting Information). There was no difference in HD tuning between conjunctive and nonconjunctive cells (Figure S7, Supporting Information); hence we included both sets of HD cells (i.e., conjunctive and nonconjunctive) for subsequent analyses. Both RS and FS HD populations were uniformly distributed in the preferred direction of HD tuning (Rayleigh test; *P* = 0.78 for FS HD cells and *P* = 0.72 for RS HD cells; Figure S5b, Supporting Information). From reconstructed tetrode tracks, it appeared that FS HD cells were enriched in layer 5, avoiding deeper layers altogether (Figure 1f).

Since the animal's head direction does not always match its movement direction,^[^ ^31^ ^]^ we further computed the movement directional tuning of the S1 cells (Figure S8a, Supporting Information). We found that a larger percentage of cells showed overlapping tuning to both head direction (HD) and movement direction (MD) (Figure S8b, Supporting Information). However, HD tuning was stronger than MD as indicated by higher mean vector length yielded by HD (Figure S8c, Supporting Information).

### 2.2 Sharply Tuned Fast‐Spiking HD Cells

**Figure 2:**
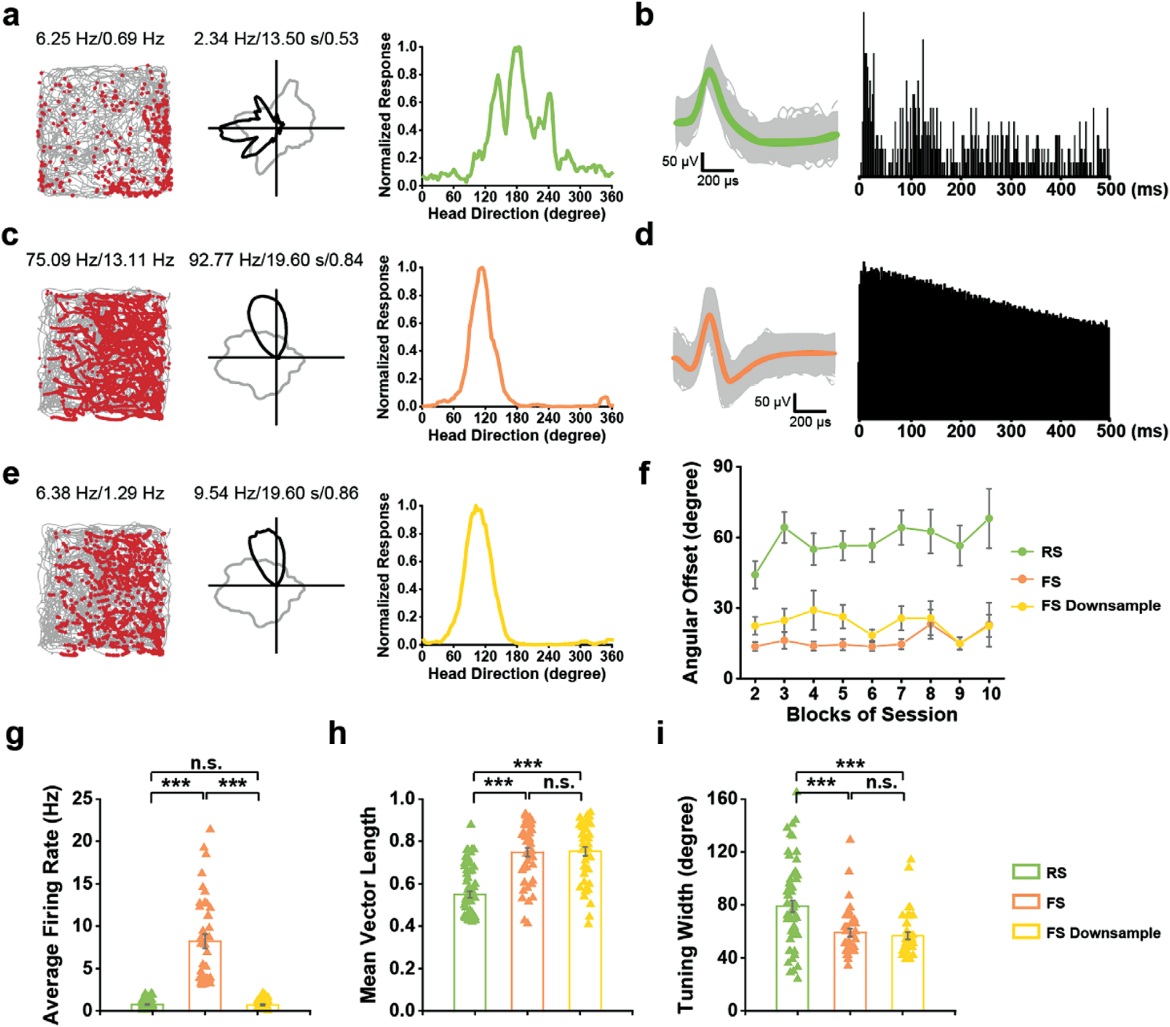
Sharper tuning of S1 fast‐spiking head direction cells. a,c) Two representative examples of S1 a) RS and c) FS HD cells. From left to right, trajectory (grey line) with superimposed spike locations (red dots; left), HD tuning curves (black) plotted against dwell‐time (grey; middle), and tuning curves from normalized firing rate (right) are presented. Peak firing rate (fr), mean fr, peak angular fr, peak dwell time and mean vector length for each representative head direction cell are labeled at the top of the panels. b,d) Left, waveforms from the representative RS HD cell (green; left in b) displays a longer spike duration than that of the representative FS HD cell (orange; left in d). Mean waveforms (colored) with superimposed spikes (grey) are shown. Right, representative autocorrelogram from the RS HD cell (right panel; b) shows a faster decay than that of the FS HD cell (right panel; d). e) Downsampled data for the FS HD cell depicted in (c). f) Angular offset of the preferred direction of HD tuning. Each session is divided into ten two‐minute blocks. The angular deviations are calculated by dividing the preferred direction of blocks #2‐10 against that of block #1. The absolute angular deviations are then averaged across cells. Data are shown in mean ± s.e.m. g) Comparable mean firing rates between RS HD cells and downsampled FS HD cells. h) Mean vector length of FS HD cells (orange, *n* = 45) as well as downsampled FS HD cells (yellow, *n* = 45) is significantly higher than that of RS HD cells (green, *n* = 60). The mean vector length of FS HD cells does not differ between raw and downsampled data. i) RS HD cells have broader tuning than FS HD cells and downsampled FS HD cells. Data are means ± s.e.m.; Mann‐Whitney *U* test, n.s., not significant; ***P < 0.001. green, RS; orange, FS; yellow, down‐sampling FS.

FS tuning to HD has been described as being weak within the canonical HD circuit,^[^ ^32^, ^33^, ^34^ ^]^ and only one single study to date has reported FS HD tuning comparable to RS HD cells in the hippocampus.^[^ ^35^ ^]^ Therefore, we sought to examine how HD tuning varied between RS and FS HD cells. Remarkably, we found that RS HD cells had broader tuning (**Figure** **2**a,b) than FS HD cells (Figure 2c,d). Since FS HD cells had higher firing rates to potentially inflate HD tuning measures, we downsampled FS firing by randomly omitting spikes from the raw spike train to match the mean firing rates of RS HD cells (Figure 2g) and tested if the sharper FS HD tuning would persist (e.g., Figure 2e). Downsampling did not change the angular stability over time (Figure 2f), the mean vector length (Figure 2h), and the tuning width (Figure 2i) of FS HD cells and all these measures remained significantly higher than RS HD cells. Downsampling FS firing rates to match the peak firing rates (rather than the mean firing rates) of RS HD cells also did not change the sharper directional tuning of FS HD cells relative to RS HD cells (Figure S9, Supporting Information). These data collectively showed FS HD cells were better tuned than their RS HD counterparts, independent of their difference in firing rate.

### 2.3 Stable Tuning of FS HD Cells in Darkness

**Figure 3:**
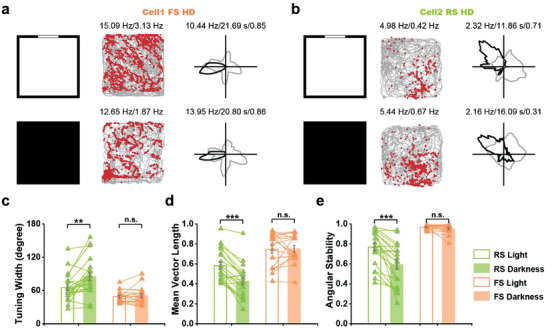
Directional tuning of RS and FS HD cells in darkness. a,b) Spatial representations of a) FS and b) RS HD cells under light and dark conditions. From left to right: schematic of experiment; trajectory (grey line) with superimposed spike locations (red dots); and HD tuning curve (black) plotted against dwell‐time (gray). Peak firing rate (fr), mean fr, peak angular fr, peak dwell time, and mean vector length for each representative HD cell are labeled at the top of the panels. c–e) Tuning width, mean vector length, and angular stability of RS and FS HD cells under light and dark conditions. Data are shown in mean ± s.e.m. *n* = 21 (RS) and 18 (FS), Wilcoxon's signed‐rank test, n.s., not significant; **P < 0.01, ***P < 0.001.

The majority of previously reported HD cells are affected under dark conditions.^[^ ^36^ ^]^ To evaluate the influence of the darkness on S1 FS and RS HD cells, we compared the effect of total darkness on HD tuning. A total of 36 sessions from seven rats were tested under light and dark conditions, among which two HD cells were co‐recorded in three sessions. FS HD cells maintained their directional tuning in darkness (**Figure** **3**a,c–e, and Figure S10, Supporting Information, *n* = 18) while RS HD cells displayed increased tuning width, decreased mean vector length and reduced angular stability from light to darkness (Figure 3b,c–e, *n* = 21). The decrease of mean vector length in RS HD cells from light to darkness was instant (Figure S11c,d, Supporting Information), while mean vector length remained stable for FS HD cells in total darkness (Figure S11a,b, Supporting Information). Moreover, the tuning of the FS HD cells remained stable across different geometric shapes (Figure S12a–d, Supporting Information) while RS HD cells exhibited less stable directional tuning (Figure S12e–h, Supporting Information).

### 2.4 Weakly Theta‐Rhythmic FS HD Cells

**Figure 4:**
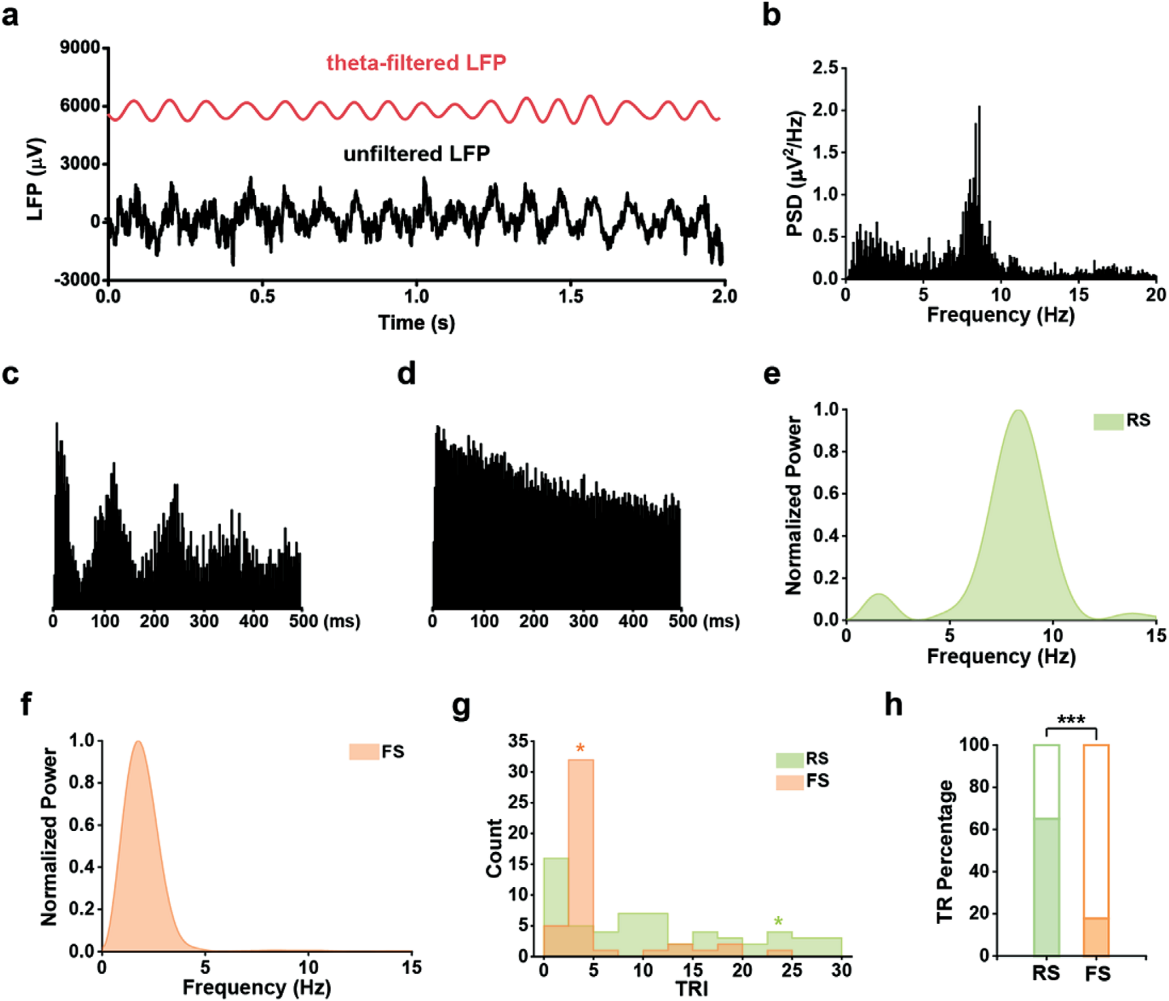
Fast‐spiking head direction cells show little theta rhythmicity. a) Prominent theta oscillations in the rat S1 during locomotion. The unfiltered signal is in black and the theta‐filtered (4–11 Hz) signal is in red. b) Power spectral density (PSD) for the whole recording session shown in (a). c,d) Two representative spike‐time autocorrelograms of c) RS and d) FS HD cells. The RS HD cell shows clear theta rhythmicity. e, f) The power spectrum of spike‐time autocorrelograms of the representative e) RS and f) FS HD cells, respectively. g) Distribution of theta rhythmicity index (TRI) of RS and FS HD cells. The asterisks label the TRI value of the representative HD cells in (c) and (d). h) The fraction of RS HD cells showing theta rhythmicity (39/60) was significantly higher than that of FS HD cells (8/45) (*χ* ^2^‐test; ***P < 0.001). Filled portions of the bars represent the proportions of theta‐rhythmic cells and unfilled portions represent non‐theta‐rhythmic cells.

Theta oscillations in the limbic system have been implicated in the integration of spatial inputs.^[^ ^37^ ^]^ However, previous studies have suggested that theta rhythmic firing of HD cells, in general, appears to be location‐ and cell type‐dependent.^[^ ^32^, ^34^, ^35^, ^38^, ^39^, ^40^, ^41^ ^]^ Here, we sought to characterize theta rhythmic firing of HD cells in the S1. First, theta oscillations could be detected in the S1 (**Figure** **4**a,b), consistent with theta‐band frequency activity in the primary somatosensory cortex reported previously.^[^ ^42^, ^43^ ^]^ To assess the possible volume‐conducted effect,^[^ ^44^ ^]^ we rederived the local field potential (LFP) by referencing the signal against that on a neighboring electrode.^[^ ^45^ ^]^ Theta oscillation was not abolished by LFP reference and a prominent peak at theta band could still be detected from the power spectra density (PSD) plot (Figure S13, Supporting Information). However, additional recordings with silicon probes and current source density analysis^[^ ^46^ ^]^ are essential to further verify the source of theta oscillation in the S1. Across all recording sessions (*n* = 295), theta power was significantly higher during active running than immobility (Wilcoxon's signed‐rank test, ***P < 0.001, Figure S14, Supporting Information).

A representative autocorrelogram of theta rhythmic RS HD cell is shown in Figure 4c and a representative autocorrelogram of a nontheta rhythmic FS HD cell is shown in Figure 4d. Power spectra of the autocorrelograms from RS HD cells had peaks at 7.5 Hz (Figure 4e), whereas a low frequency (<2 Hz) peak predominated FS HD cell autocorrelograms (Figure 4f). A subset of RS HD cells and a very few FS HD cells exhibited high theta power in their spike‐time autocorrelogram spectra (i.e., theta rhythmic index; TRI) and were deemed to be theta rhythmic (Figure 4g). The proportion of RS HD cells that showed theta rhythmicity was significantly higher than that of FS HD cells (65% vs 17.8%, *χ* ^2^‐test; ***P < 0.001, Figure 4h). In addition, the percentage of theta‐rhythmic FS HD cells was significantly lower than that of the FS cells, while the percentages of theta‐rhythmic cells for RS HD cells and RS cells were comparable (*χ* ^2^‐test; FS HD cells versus FS cells, ***P < 0.001; RS HD cells versus RS cells, *P* = 0.68).

Using an alternative maximum likelihood estimation approach,^[^ ^47^ ^]^ we demonstrated convergent evidence that a higher percentage of RS HD cells were theta rhythmic (Figure S15a–c, Supporting Information) and showed larger amplitude of theta oscillation (Figure S15d, Supporting Information), corroborating with our analysis based on binned spike times described above. Consistent with our findings here that RS HD cells displayed more theta rhythmicity, a higher percentage of RS HD cells also showed theta‐phase locking to the S1 theta band‐filtered LFP compared to their FS counterparts (Figure S15e–h, Supporting Information). To further assess whether FS HD cells exhibiting lower theta rhythmicity was a general feature of S1 FS neurons, we quantified theta rhythmicity of FS and RS cells for the entire population. Similar to FS HD and RS HD cells, we found that in general, FS cells exhibited less theta rhythmicity than RS cells in the S1 (Figure S5d,e, Supporting Information).

### 2.5 Bursting in FS HD Cells

**Figure 5:**
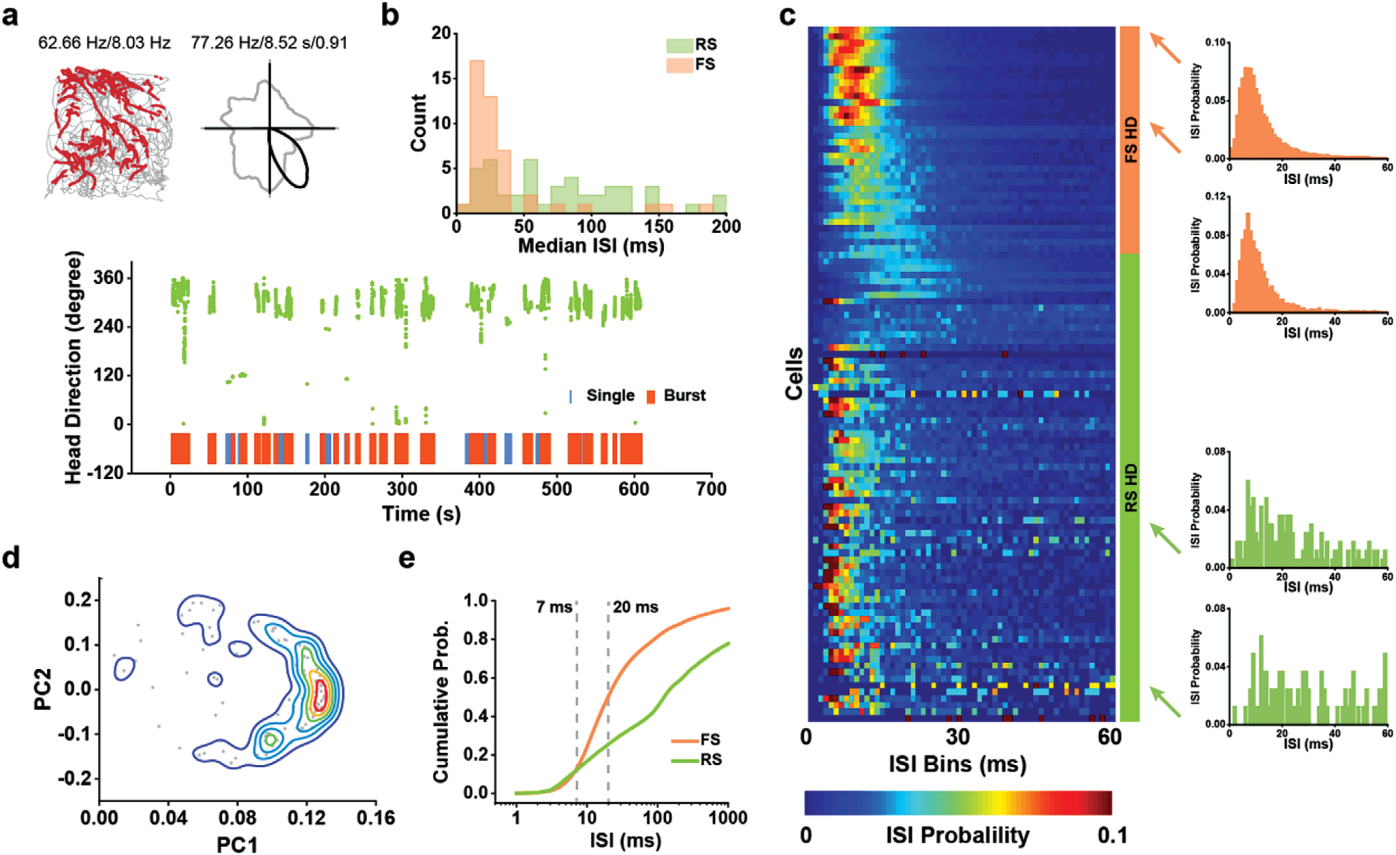
Fast‐spiking head direction cells fire in bursts. a) A representative example of a bursting FS HD cell. Trajectory (gray line) with superimposed spike locations (red dots) and head direction tuning (black) against dwell‐time plot (grey) are shown. Peak firing rate (fr), mean fr, peak angular fr, peak dwell time, and mean vector length for each representative HD cell are labeled at the top of the panels. The raster plot of the spike train (lower panel) from the FS HD cell and the corresponding HD (green dots). FS HD cell fires trains of bursts when the animal's HD matches the cell's preferred direction. b) Histogram showing distribution of median ISIs for RS and FS HD cells. c) ISIs histograms (left) of pooled FS and RS HD cells, sorted by mean firing rate. Representative ISIs histograms of two somatosensory FS HD cells (upper right, orange) and two RS HD cells (lower right, green). d) A principal component analysis (PCA) is computed based on the ISIs distribution. The scatter plot shows the first two principal components (PC1 and PC2) with 2D kernel smoothed density estimate in contour lines. e) Cumulative probability distribution of ISIs for FS and RS HD cells.

Bursting activity has been shown to encode distinct spatial signals.^[^ ^48^, ^49^ ^]^ We found that FS HD cells exhibited bursting activity when the animals’ heads were oriented in their preferred direction (**Figure** **5**a). The interspike intervals (ISIs) histogram revealed distinct temporal discharge patterns for RS and FS HD cells (Figure 5b,c). Consistent with previous studies,^[^ ^48^, ^49^ ^]^ the first two principal components of the ISI probability distribution distinguished RS and FS cells (Figure 5d). The bursting activity of FS HD cells was also reflected in the cumulative probability distributions (Figure 5e). Compared to RS HD cells, FS HD cells fired with a higher probability with ISIs between 7 and 20 ms. To directly compare the bursty properties of RS and FS cells, we computed the frequency of burst events, mean spike number in each burst, and mean burst duration for both populations (Figure S16a–c, Supporting Information). We found that both frequency of burst events and mean spike number in each burst of FS HD cells were significantly higher than the RS counterpart (Figure S16a,b, Supporting Information, Mann‐Whitney *U* test, ***P < 0.001). Meanwhile, the duration of burst events for FS HD cells was significantly longer than the RS counterpart (Figure S16c, Supporting Information, Mann‐Whitney *U* test, ***P < 0.001). Since randomly omitting spikes from the raw spike train of FS HD cells disrupted the temporal firing patterns, we found that downsampling FS HD cells to match the mean firing rates of RS HD cells would significantly disrupt the bursty firing patterns of FS HD cells (Figure S16d,e, Supporting Information).

To compare the sharpness of HD tuning between single action potentials (APs) and bursts,^[^ ^26^ ^]^ we separated the spike trains of each RS and FS HD cell into single APs and bursts (Figure S17, Supporting Information). The mean vector length of bursts was significantly higher than that of single APs for both RS and FS HD cells (Figure S17c,f, Supporting Information). Thus, bursty firing sharpens HD tuning in the S1. Moreover, the mean vector length of single APs and bursts of FS HD cells were both significantly higher than those of RS HD cells (*n* = 45 and 60, respectively, Mann‐Whitney *U* test, ***P < 0.001).

### 2.6 Somatosensory Angular Head Velocity Cells

The generation of HD tuning is believed to involve AHV cells in ring attractor models.^[^ ^9^, ^50^ ^]^ A recent report showed that AHV cells can be found alongside HD cells in the neighboring motor cortex.^[^ ^51^ ^]^ Therefore, we hypothesized AHV cells should be present in the S1.

**Figure 6:**
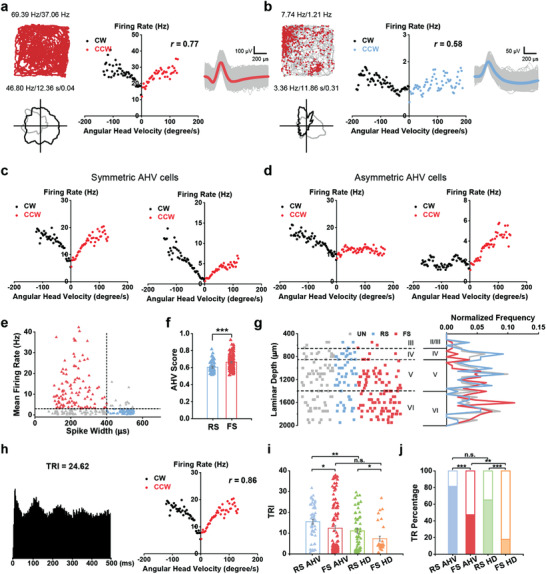
Somatosensory regular‐spiking (RS) and fast‐spiking (FS) angular head velocity cells. a) A representative FS AHV cell. Trajectory (grey line) with superimposed spike locations (red dots; top), and HD tuning curves (black) plotted against dwell‐time (grey; bottom). Peak firing rate (fr), mean firing fr, peak angular fr, peak dwell time and mean vector length for each representative AHV cell are labeled at the top of the panels. The scatter plot of binned firing rate versus angular head velocity (middle). AHV score (*r*) is labeled at the top right. Waveform of the representative FS AHV cell (right). b) The same as (a) but for a representative RS AHV cell. c) Two examples of symmetric FS AHV cells. d) Two examples of asymmetric FS AHV cells. e) Distribution of the mean firing rate versus peak‐to‐trough duration (spike width) defines three classes of AHV cells: RS (blue, *n* = 54), FS (red, *n* = 108) and unclassified (grey, *n* = 80). f) The averaged AHV score of FS AHV cells is substantially higher than that of RS AHV cells. Data are shown in mean ± s.e.m. *n* = 54 (RS) and 108 (FS), Mann‐Whitney *U* test, *P < 0.05, ***P < 0.001. g) Left, the reconstructed depth distribution of recorded AHV cells. Dashed lines delineate putative layer borders. Right, the corresponding density plot. h) Representative example of somatosensory theta‐rhythmic FS AHV cell. Left, the spike‐time autocorrelogram; Right, the scatter plot of the firing rate versus the angular velocity of the representative AHV cell. Theta‐rhythmicity index (TRI) and AHV score (*r*) are labeled at the top of the panels. i) Comparison of TRI among FS AHV cells (red), RS AHV cells (blue), RS HD cells (green) and FS HD cells (orange) (Mann‐Whitney *U* test, n.s., not significant; *P < 0.05; **P < 0.01; ***P < 0.001). j) The ratio of theta‐rhythmic FS AHV cells (51/108, 47.2%) is significantly higher than that of FS HD cells (8/45, 17.8%) but lower than that of RS AHV cells (44/54, 81.5%), which showed a similar theta‐rhythmic proportion to RS HD cells (39/60, 65%). *χ* ^2^‐test; n.s., not significant; **P < 0.05; **P < 0.01; ***P < 0.001. Filled portions of the bars represent the proportions of theta‐rhythmic cells and unfilled portions represent non‐theta‐rhythmic cells.

By calculating the first derivative of the head direction time samples using a bin size of 3° s^‐1^ and a five‐point running average,^[^ ^52^ ^]^ a total of 501/2112 putative single units recorded from the S1 were classified as AHV cells (e.g., **Figure** **6**a,b and Figure S18a, Supporting Information). Both symmetrical (*n* = 242) and asymmetrical (*n* = 259) AHV cells were found in the S1 (Figure 6c,d and Figure S19, Supporting Information). We further compared the effect of bin size and smoothing on the AHV score. We found that using 3° s^‐1^ or 6° s^‐1^ as bin size did not alter the distribution of the AHV scores of all recorded units (Figure S18b, Supporting Information). In contrast, smoothing could impact the distribution of the AHV scores (Figure S18c, Supporting Information).

As with HD cells, we further extended our symmetrical AHV cell characterization with RS and FS AHV classification (Figure 6e). Almost half of the AHV cells were FS cells (Figure S19, Supporting Information), which also had significantly higher AHV scores than RS AHV cells as a group (Figure 6f). Notably, 8% of the AHV cells showed conjunctive feature for HD (Figures S19 and S20, Supporting Information). Unlike the pattern seen for HD cells, RS AHV cells appeared to distribute across layers 4–6 while FS AHV cells were more frequently encountered in layers 5 and 6 (Figure 6g). A large majority (44/54, 81.5%) of RS AHV cells and about a half (51/108, 47.2%) of the FS AHV cells were found to be theta rhythmic (Figure 6h–j). Of note, a larger proportion of FS AHV cells were found to exhibit theta rhythmicity than FS HD cells (Figure 6i,j), while the proportions of theta‐rhythmic RS AHV cells and RS HD cells were similar (Figure 6i,j).

### 2.7 Sparse Putative Monosynaptic Connections Involving FS HD Cells

**Figure 7:**
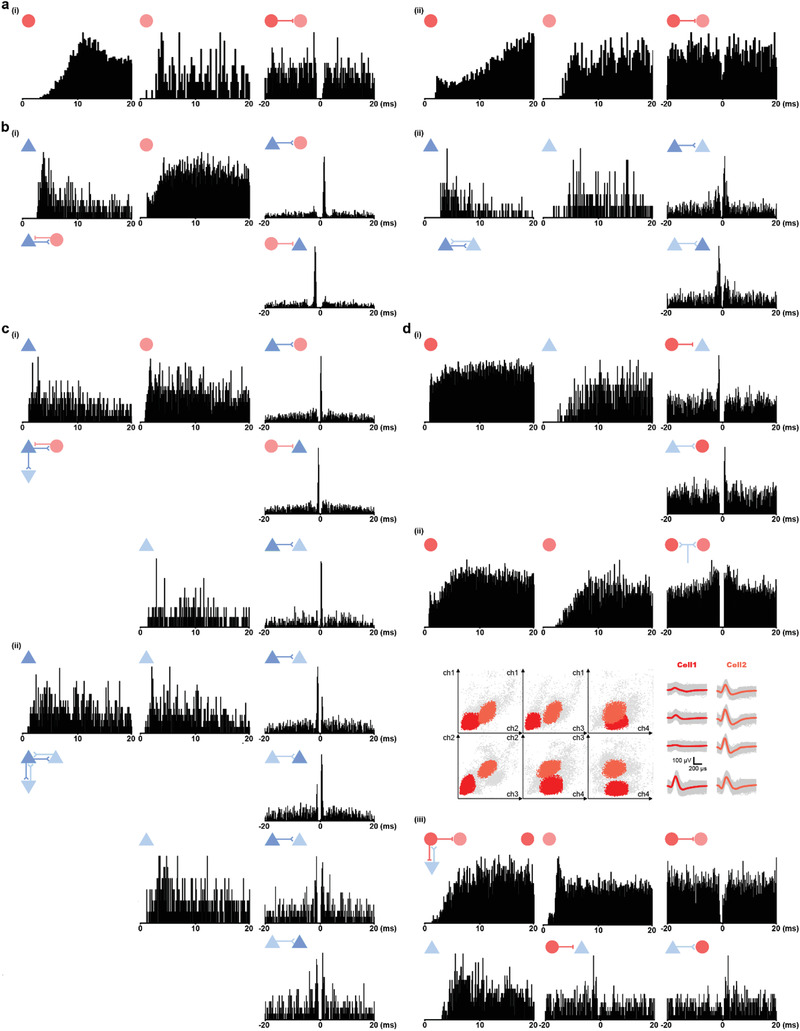
Putative monosynaptic connections between HD/AHV cells and simultaneously recorded neurons. a) Two representative monosynaptic inhibitory connections of FS HD cells with other FS cells as revealed by the spike‐time cross‐correlogram. Left, autocorrelogram of the reference cell; Middle, autocorrelogram of the target cell; Right, cross‐correlogram. Color‐coded triangles and circles represent excitatory (blue) and inhibitory (red) cells, respectively. Hypothesized synaptic connectivity is indicated at the top left of each pair. b) Two representative monosynaptic connections of RS HD cells as revealed by the spike‐time cross‐correlogram. For the reciprocal connections, two cross‐correlograms in two directions are shown. c) Putative monosynaptic connections between RS AHV cells and simultaneously recorded neurons. d) Putative monosynaptic connections between FS AHV cells and simultaneously recorded neurons (i). For the common input‐drive of FS AHV cells (ii), cluster diagrams are shown to indicate two separate cells.

Given the apparent layer‐specific distribution of HD and AHV cells in a cell‐type dependent manner, we further sought to identify putative monosynaptic connections^[^ ^48^, ^53^, ^54^ ^]^ for a better understanding of functional links between HD cells, AHV cells, and other cells in the S1. We found a small number of RS HD cells made local excitatory (and sometimes reciprocal) connections to the simultaneously recorded RS and FS cells (29/498 pairs; 5.8%; **Figure** **7**b). Notably, there were sparse putative monosynaptic connections between FS HD cells from simultaneously recorded 4/322 pairs (1.2%; Figure 7a). The spike‐time cross‐correlogram of two simultaneously recorded FS HD cells from the same tetrode showed no detectable synaptic connection (e.g., Figure S21, Supporting Information). Comparable to RS HD cells, 5.3% of simultaneously recorded cell pairs (24/453) involving RS AHV cells made excitatory connections with both RS and FS cells—mainly with other AHV cells (Figure 7c). A large proportion of cell pairs (7.3%; 46/629) involving FS AHV cells were found to make inhibitory connections to RS and FS cells, including those that were tuned to AHV themselves (Figure 7d). We also detected pairs of putative common input‐drive of FS AHV cells (Figure 7d‐ii).

## 3 Discussion

Our previous report showed many spatial cell types can be found in the S1 of freely foraging rats, including HD cells.^[^ ^23^ ^]^ In this study, we further characterized HD signaling in the rat S1 and demonstrated their coexistence of AHV signaling. In contrast to the rest of the brain where excitatory cells exhibit HD tuning, we show a disproportionately large percentage of putative interneurons (FS cells) code HD and AHV with higher precision than their putative excitatory neuron (RS cells) in the S1. Together, we present a novel configuration of HD circuitry in the S1 (Figure S22, Supporting Information) that is dissimilar to those described previously in canonical HD systems.

### 3.1 Fast‐Spiking Cells Are Tuned to Head Direction in the S1

While spatially modulated firing has been thought to be mostly confined to the hippocampal‐entorhinal system,^[^ ^55^ ^]^ the existence of HD cells outside the canonical HD/spatial circuits is not new. Head direction selectivity has been reported in the lateral dorsal thalamic nucleus,^[^ ^56^ ^]^ the striatum,^[^ ^51^, ^57^, ^58^, ^59^ ^]^ the motor cortex,^[^ ^51^, ^60^ ^]^ the visual cortex as well as the retrosplenial cortex,^[^ ^61^ ^]^ and the nucleus reuniens.^[^ ^62^ ^]^ Sharply tuned HD cells have been reported in layer 2/3 of the mouse MEC; but these cells were not classified as FS or putative interneurons.^[^ ^63^ ^]^ The only reported putative interneuron with strong HD tuning was found in the rat hippocampus, where HD cells appear to be exclusively FS cells.^[^ ^35^ ^]^ Theta and nontheta rhythmic RS HD cells could coexist in the MEC, and nonrhythmic HD cells were found more dorsally and/or in deeper layers.^[^ ^41^ ^]^ A previous study has shown that neurons in layer V of the MEC send sparse projections to the S1.^[^ ^64^ ^]^ It remains to be determined whether non‐rhythmic RS HD cells in deeper layers of the MEC contribute to HD tuning of nonrhythmic FS HD cells in the S1. Thus, our discovery of sharply tuned FS HD cells that coexist with less well‐tuned RS HD cells represents a novel and unique observation specific to the S1.

### 3.2 Fast‐Spiking Cells Are Better Tuned Than Regular‐Spiking Cells to Head Direction

The most surprising finding in our study is that ≈9% of all recorded FS are HD cells, whereas only ≈7% of the RS and unclassified cells represent HD. In fact, if we restrict HD classification to include only non‐conjunctive HD cells, FS HD cells outnumber RS or unclassified HD cells by 2:1 (9% vs 5%). Our data indicate that RS HD cells are similar to those described elsewhere,^[^ ^32^, ^34^, ^40^ ^]^ demonstrating firing characteristics of putative excitatory principal cells as well as their rhythmic firing in spike‐theta modulation. In past studies examining HD representation in the canonical HD circuit, FS cells were either excluded for spatial selectivity analysis,^[^ ^65^ ^]^ or were shown to be weakly tuned to HD^[^ ^32^, ^33^ ^]^ but better tuned to AHV.^[^ ^40^ ^]^ These studies are consistent with the previous work reported in sensory and spatial systems, showing that FS cells are poorly tuned to sensory features^[^ ^66^, ^67^, ^68^, ^69^, ^70^, ^71^, ^72^ ^]^ but may be better suited for representing speed.^[^ ^40^, ^73^, ^74^, ^75^ ^]^ However, in the cat visual cortex,^[^ ^66^, ^76^ ^]^ in the mouse auditory cortex,^[^ ^77^ ^]^ as well as in the monkey motor cortex,^[^ ^78^ ^]^ FS putative PV interneurons can exhibit stimulus selectivity comparable to pyramidal cells. Specifically, Cardin and co‐workers found all FS cells in layer 4, but not other layers, are sharply tuned to the spatial orientation of presented visual stimuli and are only marginally broader than RS cell tuning.^[^ ^66^ ^]^ In contrast to other systems where input selectivity is heterogeneous and overlapping,^[^ ^79^ ^]^ HD tuning within and across brain structures are coherent;^[^ ^80^ ^]^ thus, the divergent but homogeneous HD input may drive sharp FS HD tuning in the S1. However, our current data suggest FS HD cells are only sparsely locally connected, indicating that these FS cells may not be conventional PV^+^ neurons that have extensive local connections.^[^ ^81^ ^]^ High‐density silicon probe recordings^[^ ^53^, ^54^, ^82^ ^]^ and cell‐type‐specific tracing studies^[^ ^83^ ^]^ may provide further insight on the identity and connectivity of FS HD cells. To the best of our knowledge, no previous study has shown FS cells or putative PV^+^ interneurons displaying superior stimulus selectivity than their RS/putative excitatory cell counterparts. While the neurochemical identity of our FS HD cells remains to be elucidated, we provide the first prima facie evidence that putative PV^+^ interneurons can have sharply tuned feature selectivity.

### 3.3 Generation of HD and AHV Activity in the S1

The presence of AHV cells in the S1 opens up the possibility that HD selectivity can be locally generated in the S1. We show both symmetrical and asymmetrical AHV cells are more likely to be putative inhibitory interneurons. Along with RS/ putative excitatory HD cells, all basic components of a theorized ring attractor are present within the S1 for de novo HD signal generation.^[^ ^50^ ^]^ However, none of the examined brain areas exhibiting HD tuning is independent of the canonical HD circuit;^[^ ^51^, ^84^, ^85^, ^86^, ^87^ ^]^ It is currently unknown whether our S1 HD cells are dependent on the canonical HD circuit, since FS HD cells appear to have many different physiological properties compared to canonical HD cells. In our previous report, we suggested that S1 spatial selectivity is likely to be an efferent copy inherited from elsewhere, possibly from motor areas, given their extensive functional and anatomical connections.^[^ ^23^ ^]^ HD and AHV signals from the motor cortex^[^ ^51^, ^57^, ^60^ ^]^ may arrive through projections to layer 5,^[^ ^88^ ^]^ consistent with our observation of FS HD cells enrichment at the border of layer 4/5. Layer 4 is a focal point for the thalamocortical input via the ventral posterolateral nucleus (VPL). PV expression can be detected at the highest levels across layers 4 and 5 in mice^[^ ^28^, ^89^, ^90^ ^]^ and appears to be selectively enriched in layer 4 in rats,^[^ ^91^ ^]^ where thalamic inputs strongly target and activate PV^+^ neurons^[^ ^92^, ^93^ ^]^ in the vibrissae S1. Thalamic afferents, particularly from VPL where vestibular inputs have been reported,^[^ ^94^ ^]^ may constitute a novel alternative pathway for the generation of HD signaling in the S1.

### 3.4 Functional Significance of Sharply Tuned FS HD Cells in the S1

In the S1, the general rule of FS cells being only weakly or broadly tuned to sensory input holds true for tactile stimuli.^[^ ^24^, ^25^ ^]^ However, our data suggest HD representation is sharply tuned. What is the functional significance of having such putative inhibitory interneurons sharply tuned to HD? We reason that these sharply tuned putative interneurons might carry out the same proposed function elsewhere in the brain—to further refine principal cell HD representation. Although we have shown that RS HD representation is largely less well‐tuned than FS cells, it is entirely possible that RS HD representation may be weaker without FS HD refinement. Optogenetic or chemogenetic modulation of layer 4/5 PV^+^ neurons will be required to provide supporting evidence for sharply tuned FS HD cells to participate in improved HD tuning in principal cells. Another possible function for inhibition in the cortex is gain control. Bidirectional optogenetic modulation of PV^+^ neurons imposed gain control in visual and auditory systems instead of drastically changing principal cell tuning.^[^ ^68^, ^77^, ^95^ ^]^ Sharp tuning of putative inhibitory cells described here may relate to the need to decrease the gain of HD signal within the S1, which is compatible with the supposition that spatial representation in the S1 may relate to body parts in space, rather than the whole organism.^[^ ^23^, ^96^ ^]^ Alternatively, it has been shown in the vibrissae S1, thalamic‐mediated feedforward inhibition is key to suppressing motor contributions to somatosensation.^[^ ^97^ ^]^ In this scheme, we assume that motor inputs at least partially drive spatial responses in the S1; sharply tuned FS HD cells may provide the strong inhibition to delineate current HD (sensory) from future HD in the downstream (such as motor) structures.

Overall, we show a relatively high proportion of putative inhibitory FS cells represent HD with better precision than their putative excitatory RS counterparts. Our results challenge the prevailing view of cortical FS cell function, and how HD information is utilized in the brain. The unequivocal anatomical classification of reported FS HD cells is crucial for understanding a novel form of cortical mode of operation and (spatial) feature tuning. These findings uncover the cellular basis for sharply tuned somatosensory HD cells at the single‐cell level.

## 4 Experimental Section

### Subjects

Eleven male Long‐Evans rats (2–4 months old, 250–450 g at the time of the surgery) were used for this study. All animals were housed in groups of four prior to surgery and singly housed in transparent cages (35 cm × 45 cm × 45 cm, *W* × *L* × *H*) and maintained on a 12 hour reversed light‐dark cycle (lights on at 9 p.m. and off at 9 a.m.) after surgery. Experiments were performed during the dark phase. Rats were maintained in a vivarium with controlled temperature (19–22 °C), humidity (55–65%). and were kept at about 85–90% of free‐feeding body weight. Food restriction was imposed 8–24 h before each training and recording trial. Water was available ad libitum. All animal experiments were performed in accordance with the National Animal Welfare Act of China under a protocol approved by the Animal Care and Use Committees from both Army Medical University and Xinqiao Hospital.

### Surgery and Tetrode Placement

Rats were anesthetized with isoflurane for implant surgery. The local anesthetic lidocaine was applied to the scalp before the incision was made. Microdrives loaded with four tetrodes were implanted to target the hindlimb (S1HL), forelimb (S1FL), and shoulder (S1Sh) regions of the primary somatosensory cortex (anterior‐posterior (AP): 0.2–2.2 mm posterior to bregma; medial‐lateral (ML): 2.2–3.4 mm lateral to the midline, dorsal‐ventral (DV): 0.4/0.6–3 mm below the dura.), secured with dental cement with 8–10 anchor screws. A screw in the skull behind the eyes served as the ground electrode. After the operation, rats were given the analgesia Temgesic. Tetrodes were assembled with four 17 µm Platinum/Iridium wires (#100167, California Fine Wire Company). Tetrodes had impedances between 150 and 300 kΩ at 1 kHz through electroplating (nanoZ; White Matter LLC).

### Training and Data Collection

Behavioral training, tetrode advancement, and data recording started a week after surgery. Rats were trained to forage in a 1 m × 1 m square box with a white cue card (297 mm × 210 mm) mounted on one side of the wall. Food pellets were scattered into the arena intermittently to encourage exploration.

Each recording session lasted between 15 and 30 min to facilitate full coverage of the testing arena. Tetrodes were advanced in steps of 25 or 50 µm daily until well‐separated single units can be identified. Data were acquired by an Axona system (Axona Ltd., St. Albans, UK) at 48 kHz, band‐passed between .8–6.7 kHz and a gain of x 5–18k. Spikes were digitized with 50 8‐bit sample windows. Local field potentials were recorded from one of the electrodes with a low‐pass filter (500 Hz).

### Dark Sessions and Geometric Shapes

Darkness sessions were used to evaluate the effect of a visual landmark on an HD cell's preferred firing direction. In the darkness experiment, the light was turned off, and the cue card was removed. The shift or removal of the white cue card was performed while the rat was not present in the recording box but kept in a holding enclosure. The inter‐session interval was approximately 10 minutes for cleaning the running area and manipulating the cue card. To remove any possible olfactory cues, one cleaned the running environment with an alcohol solution and water before each session. To reduce the possible influences of the surrounding environment, one first disorientated the rats and then placed them on the running box floor in a random direction. Another set of manipulation experiments was performed to evaluate whether somatosensory HD cells can maintain their preferred head directions across different geometric shapes including one 1 m × 1 m square box, one circular arena of 1 m diameter and one 15 cm wide circular track of 1.3 m diameter.

### Spike Sorting, Cell Classification, and Rate Map

Spike sorting was manually performed offline with TINT (Axona Ltd, St. Albans, UK), and the clustering was primarily based on features of the spike waveform (peak‐to‐trough amplitude and spike width), together with additional autocorrelations and cross‐correlations.^[^ ^98^, ^99^ ^]^ During the manual cluster cutting, one always counted neurons with similar or identical waveform shapes only once across consecutive recording sessions. To confirm the quality of cluster separation, one calculated *L*‐ratio as well as isolation distance between clusters.^[^ ^26^, ^100^ ^]^ Putative fast‐spiking interneurons were classified on the basis of spike waveform and firing rate.^[^ ^101^, ^102^, ^103^, ^104^ ^]^ A mixture of two Gaussians was fitted to the distribution and the local minimum was used as the cutoff for the waveform classification.^[^ ^102^, ^105^ ^]^

Two small light‐emitting diodes (LEDs) were mounted on the headstage to track the rats’ position and head orientation via an overhead video camera with the acquisition frame rate being 50 Hz. Only spikes with instantaneous running speeds > 2.5 cm s^‐1^ were chosen for further analysis in order to exclude confounding behaviors such as immobility, grooming, and rearing.^[^ ^106^ ^]^

To classify firing fields and firing rate distributions, one divided the position data into 2.5 cm × 2.5 cm bins, and the path was smoothed with a 21 sample boxcar window filter (400 ms; 10 samples on each side).^[^ ^106^, ^107^ ^]^ Cells with > 100 spikes per session and with a coverage of >80% were included for further analyses. Maps for spike numbers and spike times were smoothed with a quasi‐Gaussian kernel over the neighboring 5 × 5 bins.^[^ ^106^ ^]^ Spatial firing rates were calculated by dividing the smoothed map of spike numbers with spike times. The peak firing rate was defined as the highest rate in the corresponding bin in the spatial firing rate map. The mean firing rate was calculated from data collected over the whole session. The spatial autocorrelation was calculated with smoothed rate maps.^[^ ^106^, ^107^ ^]^ The autocorrelograms were derived from Pearson's product‐moment correlation coefficient corrected for the edge effects and behavioral occupancy.

### Analysis of Head Direction Cells

The rat's head direction was estimated by the relative position of the LEDs differentiated through their sizes.^[^ ^17^, ^106^ ^]^ The directional tuning curve for each recorded cell was drawn by plotting the firing rate as a function of the rat's head angle, which was divided into bins of 3° and then smoothed with a 15° mean window filter (2 bins on each side). To minimize the sampling bias, one only included data if all directional bins were covered by the animal before smoothing.^[^ ^106^ ^]^

The strength of directional selectivity was calculated by computing the mean vector length from circular distributed firing rates. The chance values were determined by a shuffling process, with the entire sequence of spike trains time‐shifted between 20 s and the whole trail length minus 20 s along the animals’ trajectory. This shuffling process was repeated 100 times for each cell, generating a total of 211 200 permutations for the 2112 somatosensory neurons. This shuffling procedure preserved the temporal firing characteristics in the unshuffled data while disrupting the spatial structures at the same time. Cells were defined as head direction cells if the mean vector length of the recorded cell was larger than the 99^th^ percentile of the mean vector length in the shuffled distribution. Angular stability was computed by calculating the correlation of firing rates across directional bins generated from the first and second halves of the same trial. For computing angular stability or angular offset over time, each session was divided into multiple blocks. One computed the preferred direction for each block and the correlation of firing rates across directional bins between blocks. Tuning width was defined as the full width at half maximum (FWHM) of the HD tuning curve. To quantify the effect of firing rate on HD tuning, one randomly downsampled spike trains of FS HD cells to match either the peak firing rate or the mean firing rate of RS HD cells. Movement direction (MD) was determined as the instantaneous derivative of the animal's position and movement directional tuning was calculated in the same way as head directional tuning. The threshold for defining MD cells was determined by running a shuffling procedure performed in the same way as for HD cells. Cells with mean vector length of HD and MD passing the 99^th^ threshold of respective HD and MD shuffled data were defined as cells with overlapping tuning.

### Analysis of Angular Head Velocity Cells

The firing rate modulation by animals’ angular head velocity (AHV) was calculated as previously described.^[^ ^17^, ^52^ ^]^ Briefly, the first derivative of head direction (angular head velocity) for each time sample was computed. For each cell, the firing rate was plotted as a function of AHV in 3° s^‐1^ bin. Due to fewer samples and greater variance at higher angular velocities, one only included sample bins with total samples higher than 50 (for a total of 1 second of recording time) in order to minimize sampling bias; these samples were then smoothed by a five‐point running average. The AHV score was defined by calculating the Pearson's correlation coefficient between the angular head velocity and firing rate. Asymmetric AHV cells were defined as cells whose firing rate was positively correlated with angular head velocity in both clockwise and counter‐clockwise directions. Asymmetric AHV cells were defined as cells whose firing rate was positively correlated with angular head velocity in either the clockwise or counter‐clockwise directions but not in the opposite direction.

The threshold for defining AHV cells was determined by running a shuffling procedure performed in the same way as for HD cells. The entire sequence of spikes of a given cell was time‐shifted along the animal's path by a random interval between 20 s and the total trial length minus 20 s, with the end of the trial wrapped to the beginning and the AHV score was calculated. The shuffling procedure was repeated 100 times to generate a distribution of shuffled AHV data. Cells with AHV scores higher than the 99th percentile of the shuffled distribution were classified as AHV cells.

### Analysis of Theta Rhythmicity and Power Spectral Density

To calculate fluctuations of neural activity through the theta cycle, one filtered the rat S1 local field potentials (LFPs) to extract theta oscillations. For the low‐pass filtering, 4 and 5 Hz were selected as stopband and passband low cut‐off frequencies, respectively, while 10 and 11 Hz were selected as passband and stopband high cut‐off frequencies, respectively. Theta rhythmicity was calculated from the fast Fourier transform (FFT)‐based power spectrum of the spike‐train autocorrelation. When the mean spectral power within 1 Hz range of the theta peak within the 4–11 Hz frequency range was at least five times larger than the mean spectral power from 0 to 125 Hz (the ratio defined as theta rhythmicity index, TRI), the cell was classified as being theta rhythmic. To reduce the bias of the examination of theta rhythmicity by spike‐time autocorrelogram, one applied maximum likelihood estimation (MLE) to a parametric model of the lags.^[^ ^47^ ^]^

### Analysis of Bursty Firing Properties

One generated the interspike intervals (ISIs) histogram to examine bursting.^[^ ^49^ ^]^ ISI probability distribution was first computed for each head direction cell by binning the ISIs below 60 with 1 ms bins and normalized such that the area under the curve equals 1. Then a principal component analysis was performed on the ISI probability distributions for all the neurons and the first two principal components (PC1 and PC2) were obtained, followed by a 2D Gaussian kernel smoothed density estimate.^[^ ^108^ ^]^

### Cross‐Correlogram and Putative Synaptic Connections

One identified putative monosynaptic connections by using spike‐time cross‐correlograms as described by others.^[^ ^53^, ^54^ ^]^ Briefly, short‐latency (<4 ms) peaks with the amplitude above 5 SDs of baseline mean of the cross‐correlograms were considered as putative monosynaptic excitatory connections. Similarly, short‐latency (<4 ms) troughs with the amplitude below 5 SDs of the baseline mean of the cross‐correlogram were considered as putative monosynaptic inhibitory connections. For neuron pairs simultaneously recorded from the same electrode, the 0–1 ms bins of the cross‐correlogram were not considered since superimposed spikes corecorded on the same electrode could not be resolved by the clustering program.^[^ ^53^ ^]^

### Histology and Reconstruction of Recording Positions

At the end of the experiment, rats were euthanized with an overdose of sodium pentobarbital and perfused transcardially with phosphate‐buffered saline (PBS) followed by 4% paraformaldehyde (PFA). Afterward, the brains were removed and stored in a 4% PFA solution overnight. Each brain would then be placed in 10, 20, and 30% sucrose/PFA solution sequentially across 72 h before sectioning using a cryotome. Thirty‐micron sections were obtained through the implant region. Sections were mounted on glass slides and stained with cresyl violet (Sigma‐Aldrich). The final recording positions were determined from digitized images of the Nissl‐stained sections. Positions of each recording were estimated from the deepest tetrode track, notes on tetrode advancement with tissue shrinkage correction by dividing the distance between the brain surface and electrode tips by the last advanced depth of the recording electrodes. All electrode traces were confirmed to be located within the S1 defined by The Rat Brain Atlas.^[^ ^109^ ^]^

### Statistics

Statistical analyses were performed using SPSS statistical software (IBM SPSS Statistics 20; USA) and MATLAB (The MathWorks; USA). For comparisons between two groups, Mann‐Whitney *U* tests were applied. For paired comparisons, Wilcoxon's signed‐rank tests were used. Rayleigh test was conducted for uniformity of circular data. *χ* ^2^‐test was used for ratio comparison. Binomial test was applied for chance level tests. *P* values of < 0.05 were considered significant. n.s., not significant, *P* > 0.05, *P < 0.05, **P < 0.01, and ***P < 0.001. Data are means ± s.e.m.

## Conflict of Interest

The authors declare no conflict of interest.

## Author Contributions

X.L., B.D., and C.K.Y. contributed equally to this work. S.‐J.Z. conceived the project. X.L. and S.‐J.Z. designed the study. X.L., B.D., and S.‐J.Z. performed the experiments and collected the data. X.L. and S.‐J.Z. conducted the analyses. Z.S.C. and C.K.Y. participated in the analyses. Q.C., G.‐L.L., and Z.Z. assisted in the recordings. S.‐Q.L. and H.Y. helped with the chronic surgery. X.L., C.K.Y., and S.‐J.Z. wrote the manuscript.

## Supporting information

Supporting Information

## Data Availability

The data that support the findings of this study are available from the corresponding author upon reasonable request.
