# Supplementary material for: Sharp Tuning of Head Direction and Angular Head Velocity Cells in the Somatosensory Cortex
Source: Adv Sci (Weinh). 2022 Mar 17;9(14):2200020. doi: 10.1002/advs.202200020 (PMC9109065; doi:10.1002/advs.202200020)
Supplement: Supplementary file 1 — Supporting Information [file ADVS-9-2200020-s001.pdf]

ADVANCED SCIENCE
Open Access

Supporting Information

for *Adv. Sci.*, DOI 10.1002/advs.202200020

Sharp Tuning of Head Direction and Angular Head Velocity Cells in the Somatosensory Cortex

*Xiaoyang Long, Bin Deng, Calvin K. Young, Guo-Long Liu, Zeqi Zhong, Qian Chen, Hui Yang, Sheng-Qing Lv, Zhe Sage Chen and Sheng-Jia Zhang**

Supporting Information

Sharp tuning of head direction and angular head velocity cells in the somatosensory cortex

*Xiaoyang Long, Bin Deng, Calvin K. Young, Guo-Long Liu, Zeqi Zhong, Qian Chen, Hui Yang, Sheng-Qing Lv, Zhe Sage Chen and Sheng-Jia Zhang**

Table S1. Total number of each types of HD cells by animals.

Animal#	RS HD	FS HD	Unclassified HD
Rat# 1	6	7	7
Rat# 2	8	3	10
Rat# 3	8	4	6
Rat# 4	5	4	6
Rat# 5	7	3	1
Rat# 6	4	7	3
Rat# 7	3	0	2
Rat# 8	3	6	5
Rat# 9	2	9	2
Rat# 10	7	2	1
Rat# 11	7	0	10

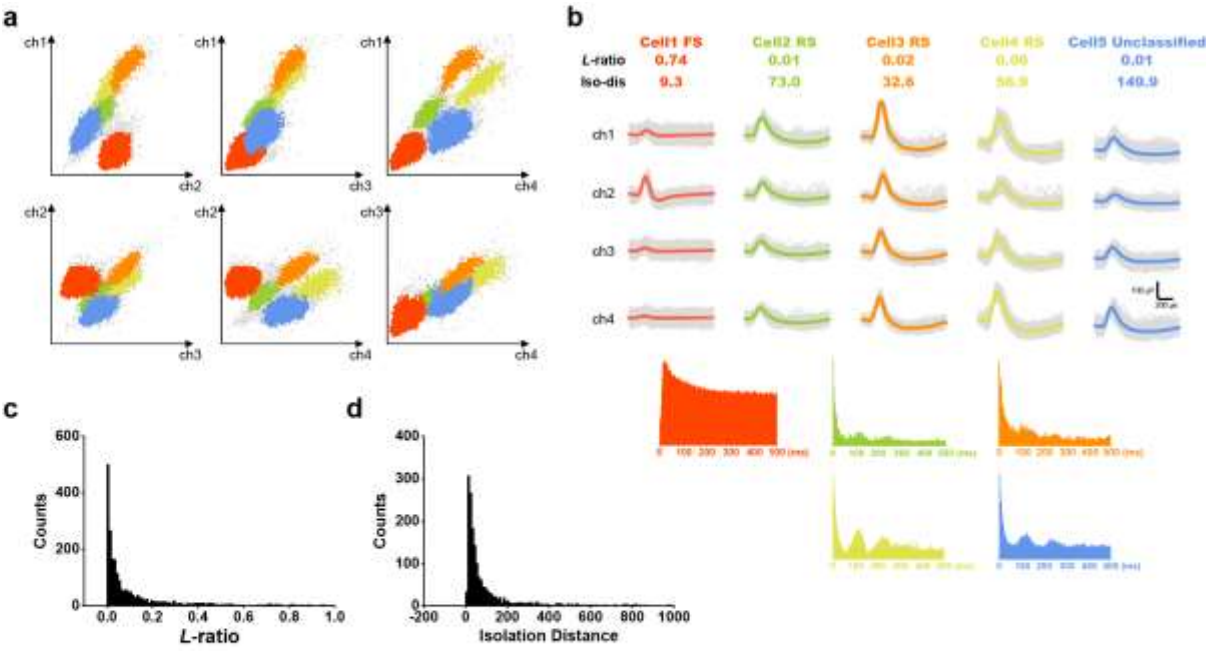

Figure S1. Cluster diagrams and waveforms recorded from the primary somatosensory cortex (S1). a) Scatter plots show the relationship between peak-to-trough amplitudes for all spikes, which are recorded on six combinations of channels from four tethered electrodes (ch1-ch4) on a specific tetrode from one representative recording session. Each dot represents a single

recorded spike. Each color indicates one well-separated cluster with grey dots indicating unclustered spikes. The cluster diagrams show spike clusters from our recordings in the S1 are well separated. b) Waveforms from one FS (red),– three RS (green, orange and yellow) and one unclassified (blue) cells in the scatter plots corresponding to the color-coded cluster in (a). Waveforms are shown for each of the four electrodes of the tetrode. *L*-ratio and isolation distance (Iso-dis) for each example cell are indicated. Spike-time autocorrelograms of the representative cells are shown below. c, d) The distribution of *L*-ratio and isolation distance for all identified somatosensory units (n = 2112).

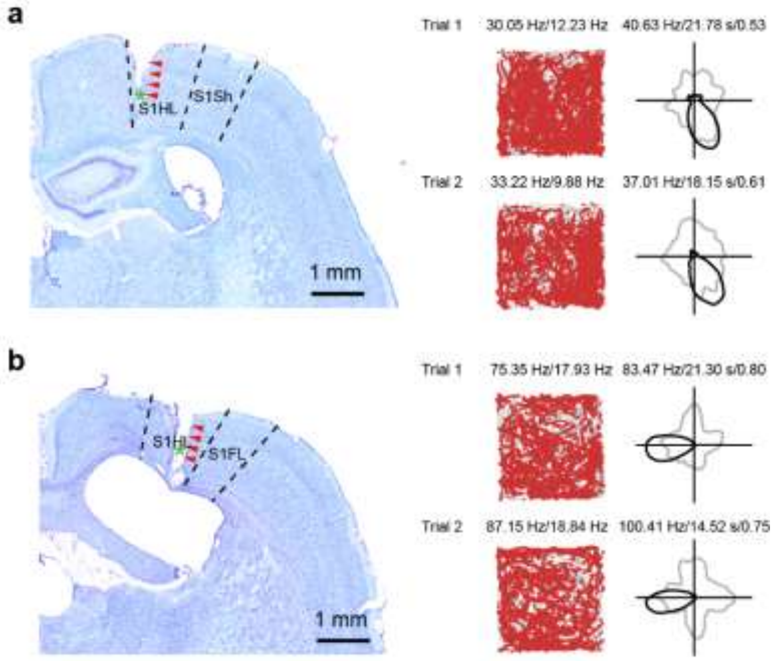

Figure S2. Electrode track and recording locations in the primary somatosensory cortex (S1). a, b) Left, Nissl-stained coronal brain sections showing recording electrode tracks (arrowheads) in the rat primary somatosensory cortex. S1HL, hindlimb region; S1FL, forelimb region; S1Sh, shoulder region. Scale bar, 1 mm. Right, spike activity from a representative FS HD cell during two trials from recording location labeled by green asterisk on the left brain section. Trajectory (grey line) with superimposed spike locations (red dots; left), and HD tuning curves (black) plotted against dwell-time (grey; right) are presented. Peak firing rate (fr), mean fr, peak angular fr, peak dwell time and mean vector length for each representative HD cell are labeled at the top of the panels.

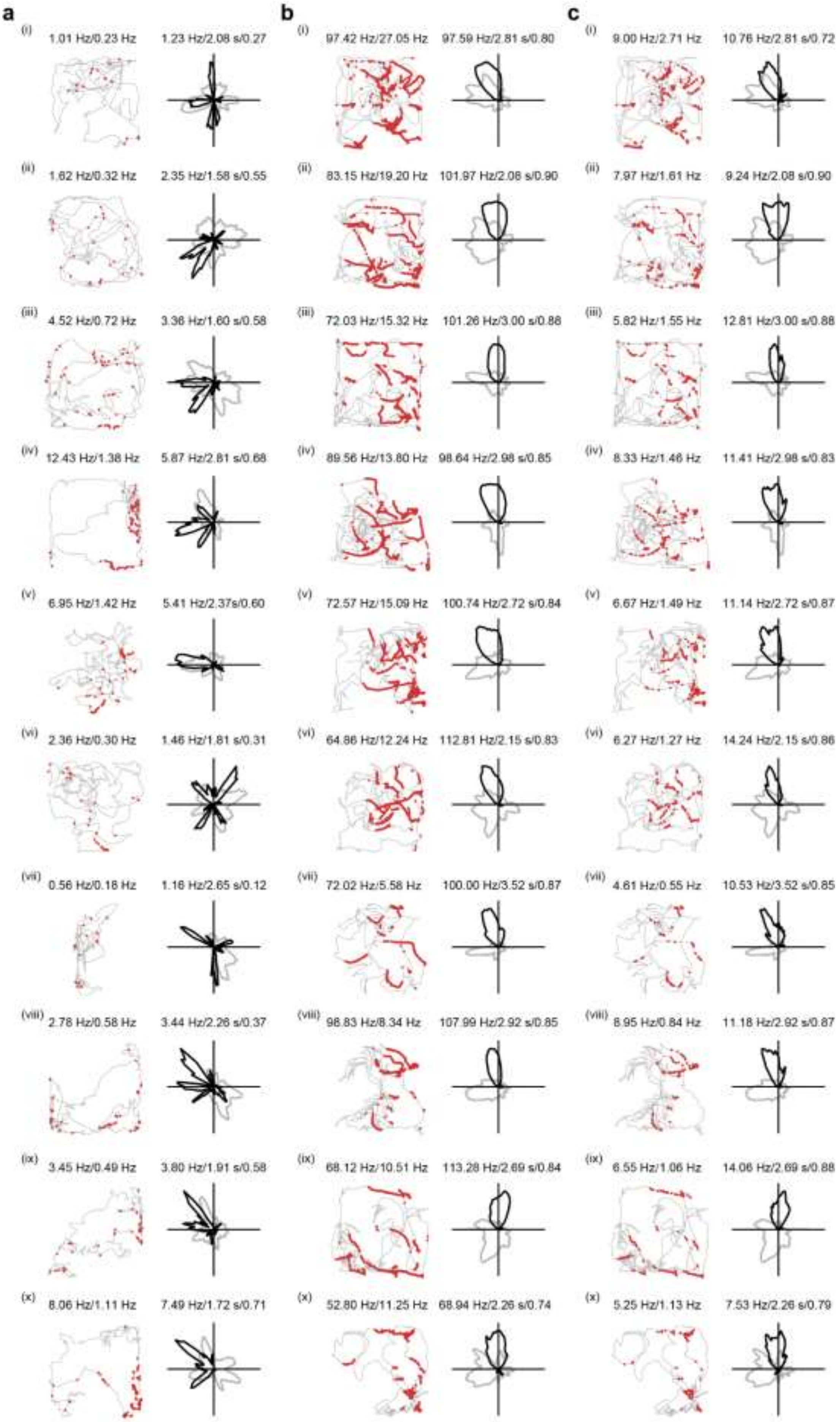

a
(i) 1.01 Hz/0.23 Hz
1.23 Hz/2.08 s/0.27
(ii) 1.62 Hz/0.32 Hz
2.35 Hz/1.58 s/0.55
(iii) 4.52 Hz/0.72 Hz
3.36 Hz/1.60 s/0.58
(iv) 12.43 Hz/1.38 Hz
5.87 Hz/2.81 s/0.68
(v) 6.95 Hz/1.42 Hz
5.41 Hz/2.37s/0.60
(vi) 2.36 Hz/0.30 Hz
1.46 Hz/1.81 s/0.31
(vii) 0.56 Hz/0.18 Hz
1.16 Hz/2.65 s/0.12
(viii) 2.78 Hz/0.58 Hz
3.44 Hz/2.26 s/0.37
(ix) 3.45 Hz/0.49 Hz
3.80 Hz/1.91 s/0.58
(x) 8.06 Hz/1.11 Hz
7.49 Hz/1.72 s/0.71
b
(i) 97.42 Hz/27.05 Hz
97.59 Hz/2.81 s/0.80
(ii) 83.15 Hz/19.20 Hz
101.97 Hz/2.08 s/0.90
(iii) 72.03 Hz/15.32 Hz
101.26 Hz/3.00 s/0.88
(iv) 89.56 Hz/13.80 Hz
98.64 Hz/2.98 s/0.85
(v) 72.57 Hz/15.09 Hz
100.74 Hz/2.72 s/0.84
(vi) 64.86 Hz/12.24 Hz
112.81 Hz/2.15 s/0.83
(vii) 72.02 Hz/5.58 Hz
100.00 Hz/3.52 s/0.87
(viii) 98.83 Hz/8.34 Hz
107.99 Hz/2.92 s/0.85
(ix) 68.12 Hz/10.51 Hz
113.28 Hz/2.69 s/0.84
(x) 52.80 Hz/11.25 Hz
68.94 Hz/2.26 s/0.74
c
(i) 9.00 Hz/2.71 Hz
10.76 Hz/2.81 s/0.72
(ii) 7.97 Hz/1.61 Hz
9.24 Hz/2.08 s/0.90
(iii) 5.82 Hz/1.55 Hz
12.81 Hz/3.00 s/0.88
(iv) 8.33 Hz/1.46 Hz
11.41 Hz/2.98 s/0.83
(v) 6.67 Hz/1.49 Hz
11.14 Hz/2.72 s/0.87
(vi) 6.27 Hz/1.27 Hz
14.24 Hz/2.15 s/0.86
(vii) 4.61 Hz/0.55 Hz
10.53 Hz/3.52 s/0.85
(viii) 8.95 Hz/0.84 Hz
11.18 Hz/2.92 s/0.87
(ix) 6.55 Hz/1.06 Hz
14.06 Hz/2.69 s/0.88
(x) 5.25 Hz/1.13 Hz
7.53 Hz/2.26 s/0.79

Figure S3. Angular stability of S1 head direction cells. a-c) Intra-session angular stability across ten two-minute blocks of a single session of RS (a), FS (b) and downsampled FS (c) HD cells from Figure 2a-c. Trajectory (grey line) with superimposed spike locations (red dots; left), and HD tuning curve (black) plotted against dwell-time (grey; right) for ten blocks (i-x) of the trials are shown. Peak firing rate (fr), mean fr, peak angular fr, peak dwell time and mean vector length for each representative HD cell are labeled at the top of the panels. Each row (i-x) represent data from a two-minute block.

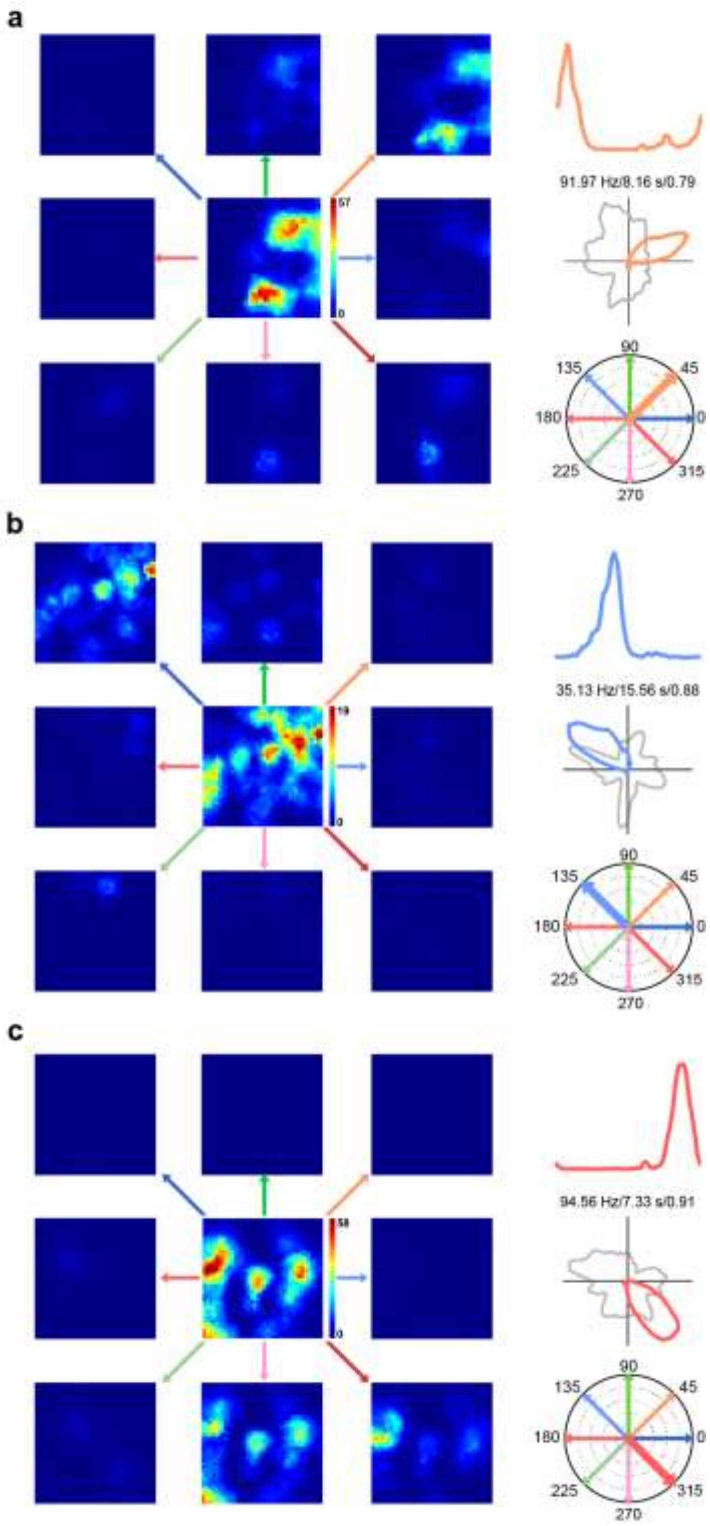

Figure S4. Head direction tuning of S1 FS HD cells is not biased by location. a-c, Three examples of S1 FS HD cells recorded from freely moving rats. Left, one spatial firing rate map of a single FS HD cell is divided into eight smoothed rate maps across all eight HD sectors segmented by 45° each. Top right, HD tuning curve. Middle right, firing rate polar plots plotted against dwell-time polar plot (grey). Peak angular firing rate, peak dwell time

and mean vector length for each representative HD cell are labeled at the top of the panels. Bottom right, circular distribution of the preferred firing direction across 360°.

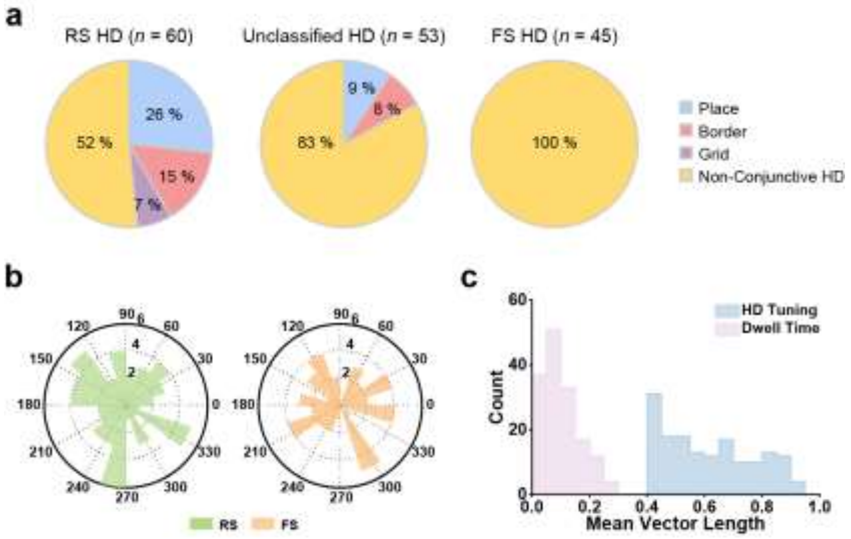

Figure S5. Classification of somatosensory head direction cells. a) Pie charts showing the proportions of fast-spiking (FS), regular-spiking (RS) and unclassified HD cells into conjunctive and non-conjunctive HD cells. b) The circular distributions of the preferred direction for two discrete RS (green) and FS (orange) groups from all eleven rats (Rayleigh test, $n = 60$ and 45, $P = 0.72$ and 0.78, respectively). c) Histograms showing the distributions of mean vector length for dwell time versus HD tuning for all identified S1 HD cells.

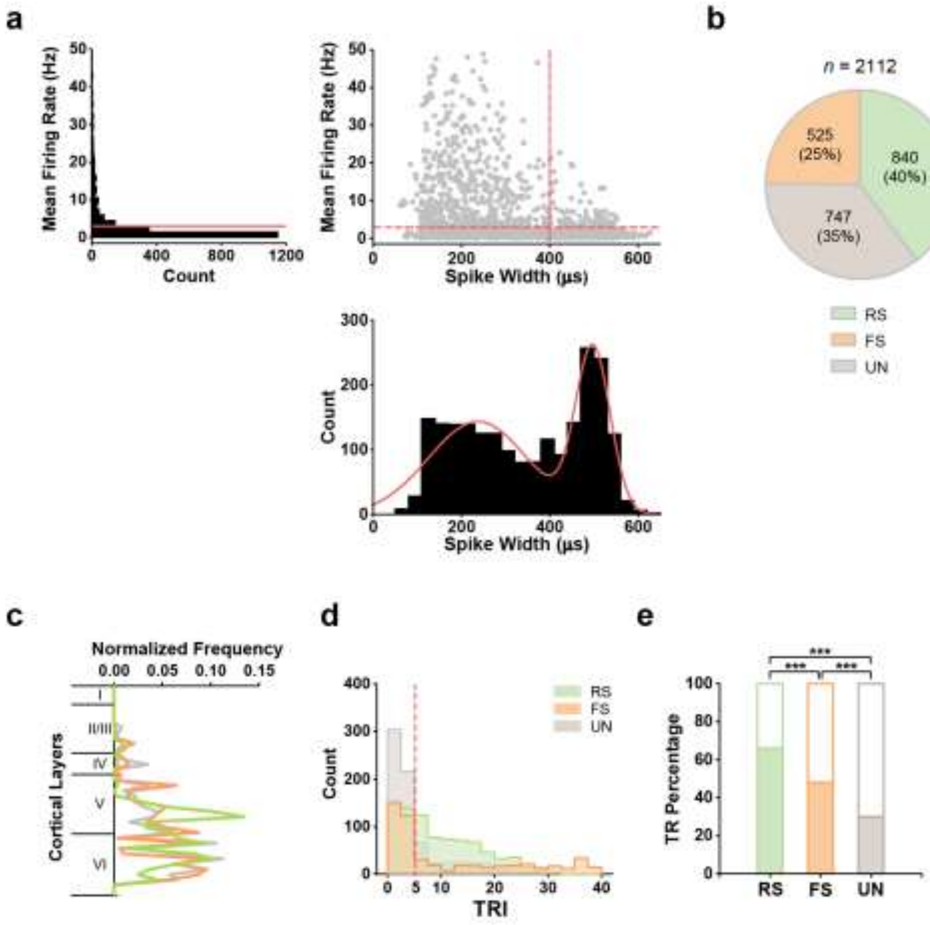

Figure S6. Cell classification and theta rhythm. a) Cell classification on the basis of waveform and mean firing rate. Top right, scatter plot of mean firing rate versus peak-to-trough waveform duration for the population of somatosensory cells. Left, histogram of mean firing rates for the whole population. Bottom, histogram of peak-to-trough spike widths for the whole population. A mixture of two Gaussians (red trace) was fitted and a local minimum (400 μs) was used as the classification threshold. b) Pie chart showing the proportion of RS, FS and unclassified (UN) cells. c) Layer density distribution for three types of cells. d) Histogram showing the theta rhythmicity index (TRI) of three types of cells. The red dashed line indicates threshold (TRI = 5) for defining a cell as being theta-rhythmic. e) Ratio of cells showing theta rhythmicity (TR) for RS, FS and UN cells (χ^2-test; ***$P < 0.001$). Filled portions of the bars represent the proportions of theta-rhythmic cells and unfilled portions represent non-theta-rhythmic cells.

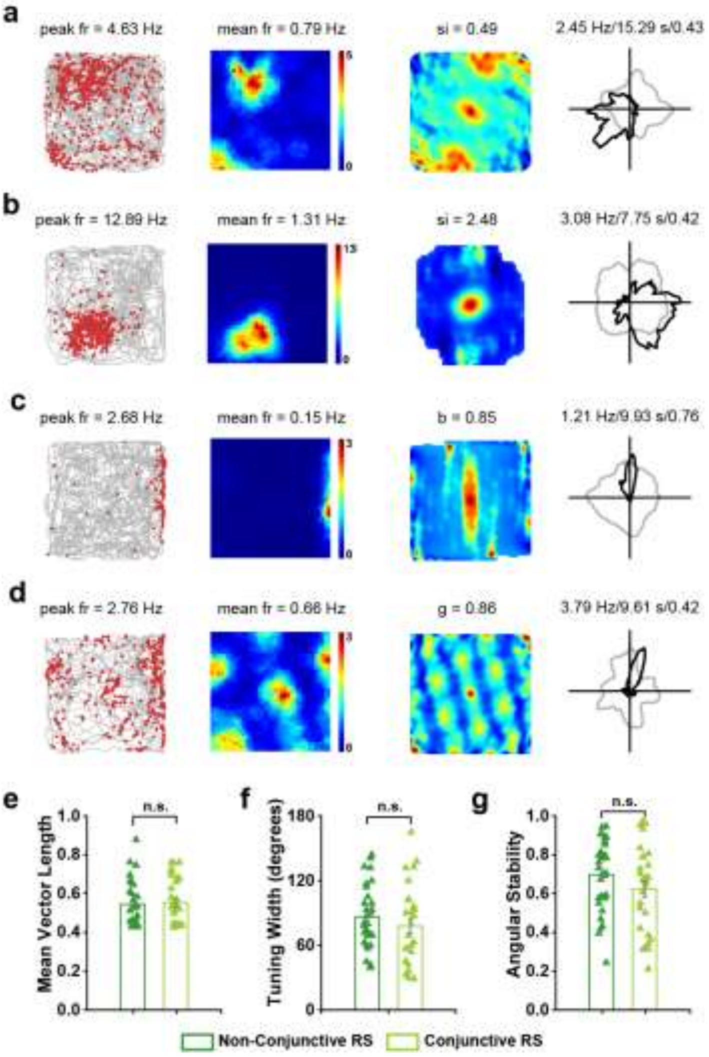

Figure S7. Conjunctive and non-conjunctive regular-spiking cells show similar head directional tuning. a-d) Example non-conjunctive RS HD, conjunctive RS HD × place, conjunctive RS HD × border and conjunctive RS HD × grid cells. From left to right: trajectory (grey line) with superimposed spike locations (red dots); smoothed rate maps; spatial autocorrelation; and HD tuning curve (black) against dwell-time (grey). Peak firing rate (fr), mean fr, peak angular fr, peak dwell time and mean vector length for each representative cell are labeled at the top of the panels. Spatial information (si), border score (b) and grid score (g) are also indicated. e) Non-conjunctive RS HD cells (dark green, $n = 31$) and conjunctive RS HD cells (light green, $n = 29$) have comparable mean vector length (Mann-Whitney U test; $P = 0.59$). f) Non-conjunctive RS HD cells show comparable tuning width as conjunctive RS HD cells (Mann-Whitney U test; $P = 0.27$). g) Angular stability within sessions for conjunctive and non-conjunctive RS HD cells is not statistically different (Mann-Whitney U test; $P = 0.16$). Data are shown in mean ± s.e.m.

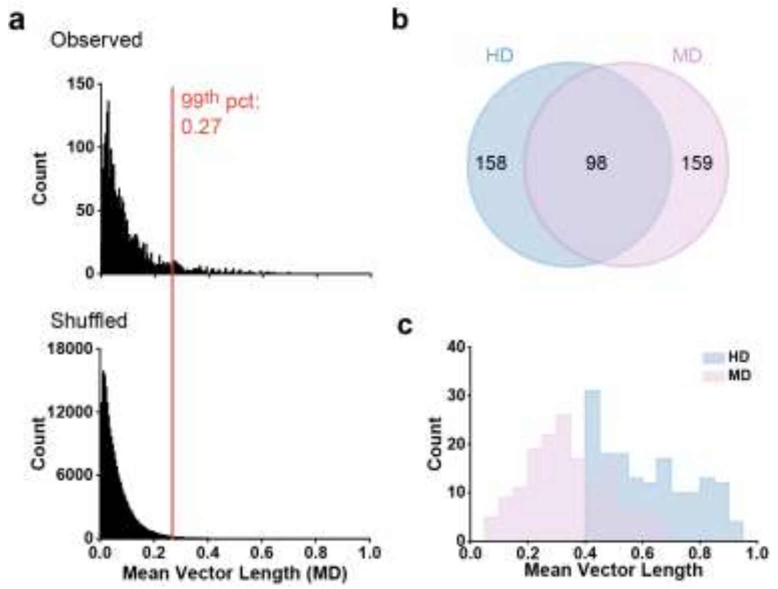

Figure S8. Movement direction tuning in the somatosensory cortex. a) Distribution of mean vector length for movement direction (MD) for the entire sample of recorded somatosensory cells. The top panel shows the distribution for observed values. The bottom panel shows the distribution for randomly shuffled data from the same population. The red line and number indicate 99th percentile for the shuffled data. b) Venn diagram showing the overlap of cells tuned to both HD and MD. c) Histogram showing the distribution of mean vector length for HD and MD for identified HD cells.

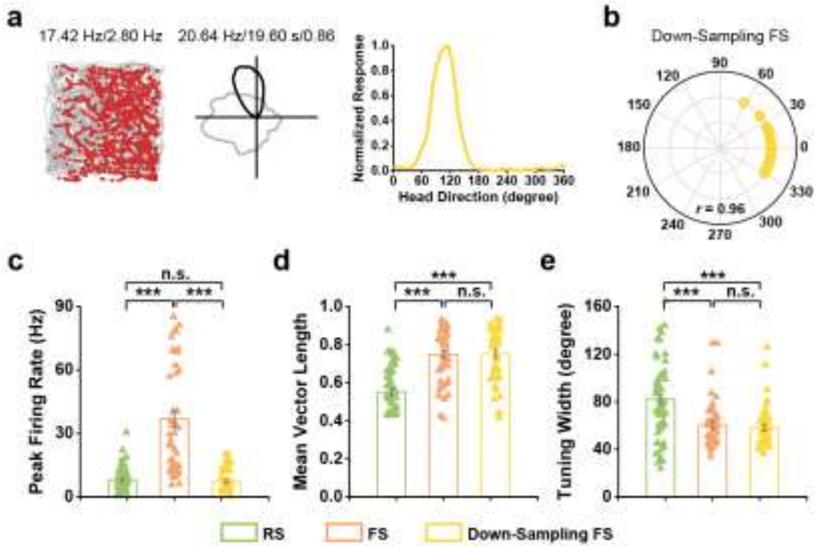

Figure S9. Downsampling FS HD cells to match the peak firing rates of RS HD cells. a) Downsampled data on the basis of peak firing rate for the representative FS HD cell depicted in Figure 2c. From left to right, trajectory (grey line) with superimposed spike locations (red dots; left), HD tuning curves (black) plotted against dwell-time (middle), and tuning curves

(right) are presented. Peak firing rate (fr), mean fr, peak angular fr, peak dwell time and mean vector length for the representative head direction cell are labeled at the top of the panels. b) Polar plot showing the differences of the preferred direction of HD tuning between the first half and second half of the same recording sessions for downsampled FS HD cells. Mean resultant length (*r*) is indicated. c) Downsampling firing rate of FS HD cells to match the peak firing rate of RS HD cells. d) Mean vector length of FS HD cells (orange, n = 45) as well as mean vector length of downsampled FS HD cells (yellow, n = 45) are significantly higher than that of RS HD cells (green, n = 60). Mean vector length of FS HD cells does not differ between raw and downsampled data. e) RS HD cells have broader tuning than FS HD cells and downsampled FS HD cells. Data are shown in mean ± s.e.m. Mann-Whitney *U* test, n.s., not significant; ***$P < 0.001$. green, RS; orange, FS; yellow, down-sampling FS.

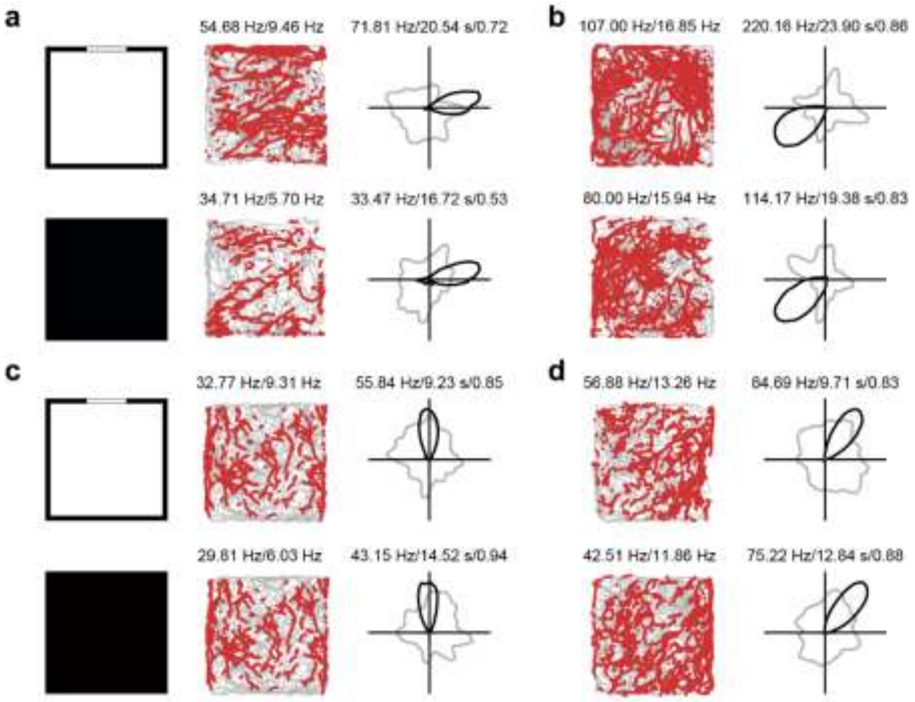

Figure S10. Preferred head directionality of fast-spiking head direction cells remain unaltered in darkness. a-d) Spatial representations of four representative FS HD cells in light and dark conditions. From left to right: schematic of manipulation; trajectory (grey line) with superimposed spike locations (red dots); and HD tuning curve (black) plotted against dwell-time (grey). Peak firing rate (fr), mean fr, peak angular fr, peak dwell time and mean vector length for each representative HD cell are labeled at the top of the panels.

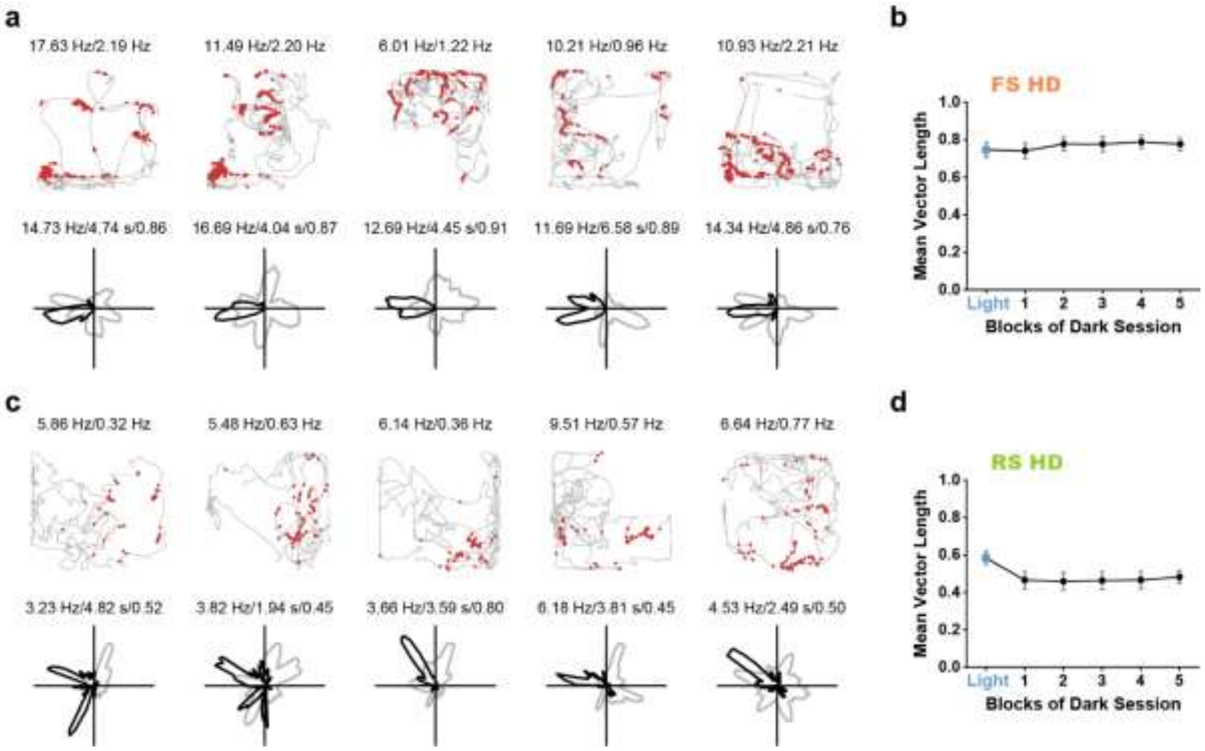

Figure S11. Head direction tuning of RS HD cells decreases instantly in total darkness. a) The session in the darkness is divided into blocks of five minutes each to show the development of HD tuning in total darkness of the representative FS HD cell in Figure 3a. From top to bottom, trajectory (grey line) with superimposed spike locations (red dots), and HD tuning curve (black) plotted against dwell-time (grey). Peak firing rate (fr), mean fr, peak angular fr, peak dwell time and mean vector length for each representative HD cell are labeled at the top of the panels. b) Stable tuning of FS HD cells from light to darkness. c) Same in (a) but for the representative RS HD cell in Figure 3b. d) Instant decrease of mean vector length of RS HD cells from light to darkness, which persists for the rest of the session in darkness. Data are shown in means ± s.e.m.

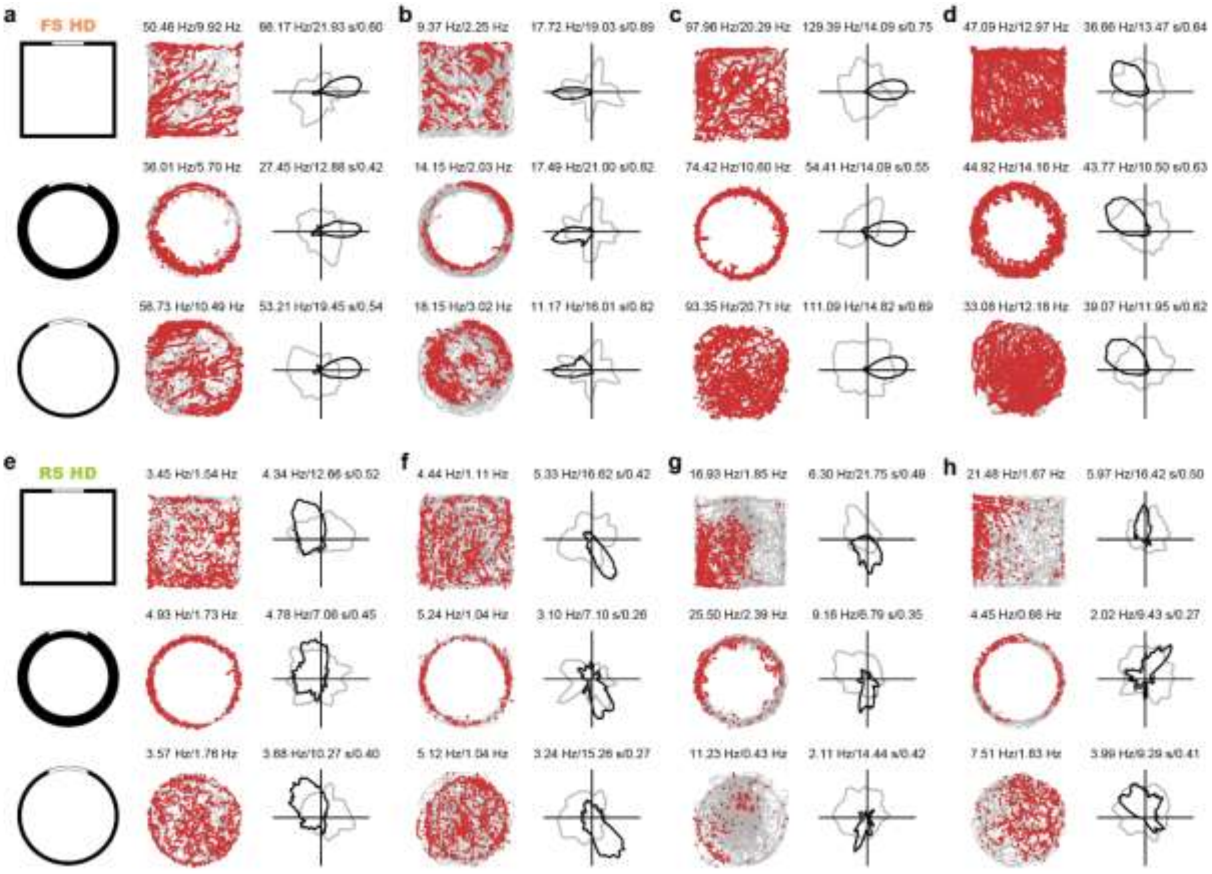

Figure S12. Maintenance of fast-spiking HD cells' preferred firing directions across different geometric shapes. a-d) Four representative FS HD cells maintaining their preferred head direction tuning across different geometric shapes. From left to right: schematic of different shapes of running box; trajectory (grey line) with superimposed spike locations (red dots); and HD tuning curve (black) plotted against dwell-time (grey). Peak firing rate (fr), mean fr, peak angular fr, peak dwell time and mean vector length for each representative HD cell are labeled at the top of the panels. e-h) Same as panels a-d, but for representative RS HD cells in different geometric shapes. Mean absolute angular deviation of preferred peak directions, square versus circular track, RS: 20.46°±5.79°, FS: 7.14°±0.73°; square versus circle, RS: 14.28°±4.04°, FS: 4.76°±1.19°, n = 5 for both RS and FS HD cells.

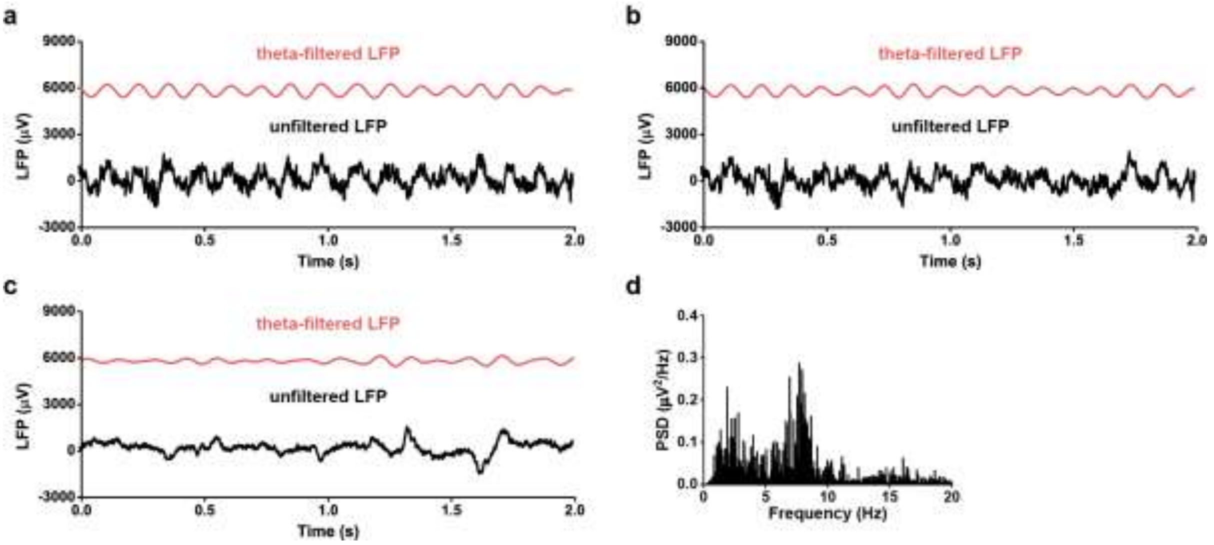

Figure S13. LFP referencing from two adjacent recording electrodes. a, b) Theta oscillations during locomotion at two adjacent electrodes in the rat S1. The unfiltered signal is in black and theta-filtered (4-11 Hz) signal is in red. c) LFP after referencing between two neighboring electrodes shown in (a) and (b). d) Power spectral density (PSD) for the whole recording session for re-referenced data shown in (c).

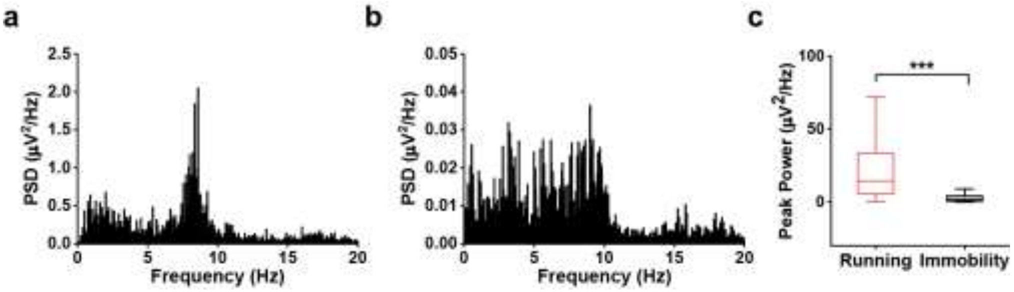

Figure S14. Strong theta oscillation in the primary somatosensory cortex during locomotion. a, b) Power spectral density (PSD) plots of the same recording session in Figure 4a during active running (a) and immobility (b). c) Peak power of theta oscillation is significantly higher during locomotion than immobility (Wilcoxon's signed-rank test, n = 295, ***P < 0.001). Data are shown in means ± s.e.m.

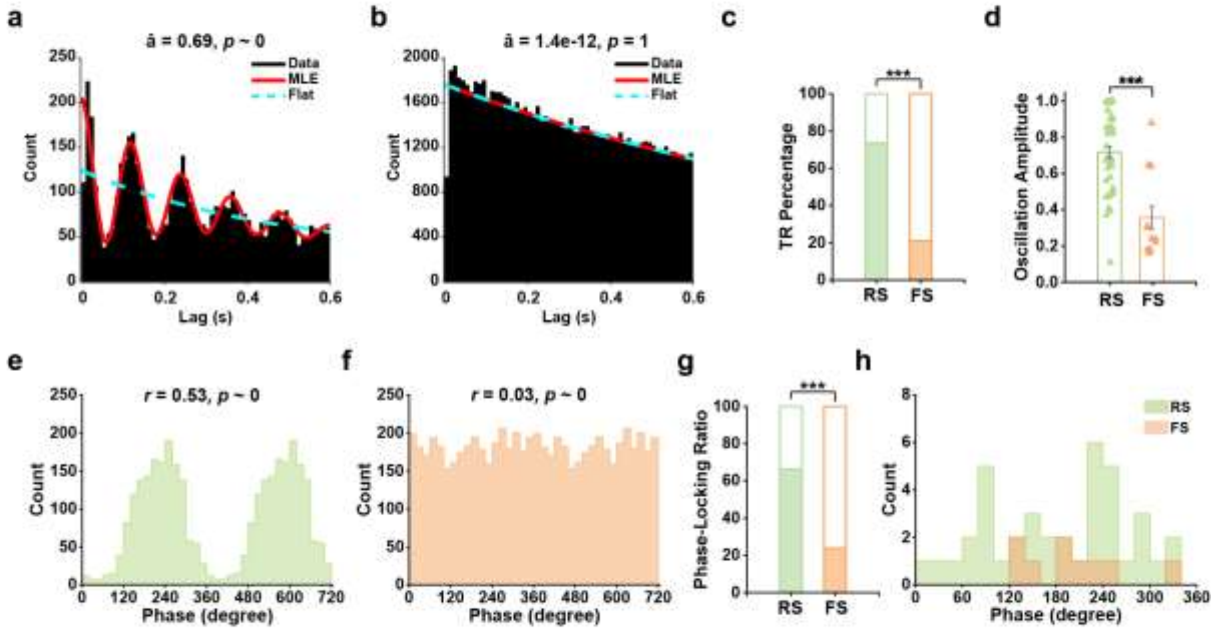

Figure S15. Maximum likelihood estimate (MLE) method for RS and FS HD cells. a, b) Theta rhythmicity estimation using the MLE approach for the examples in Figure 4c, d. The red line shows the approximated histogram from the best fit, the blue dashed line shows the best fit without rhythmicity. The amplitude of the oscillation (â) is labeled above the figure. *P*-values are the results of the likelihood-ratio test for the full fit (red line) over the non-rhythmic fit (blue dashed line). c) The proportion of FS HD cells showing theta rhythmicity (TR) was significantly lower than that of RS HD cells (χ^2-test; ***$P < 0.001$). Filled portions of the bars represent the proportions of theta-rhythmic cells and unfilled portions represent non-theta-rhythmic cells. d) The mean amplitude of oscillation of FS HD cells with theta rhythmicity was lower than that of RS HD cells (Mann-Whitney *U* test, $n = 45$ and 13, respectively, ***$P < 0.001$). Data are shown in mean ± s.e.m. e, f) Phase distributions for the representative examples in Figure 4c, d. g) The ratio of phase-locking FS HD cells versus RS HD cells. Filled portions of the bars represent the proportions of phase-locking cells and unfilled portions represent non-phase-locking cells. h) Distributions of preferred phases for FS HD cells and RS HD cells.

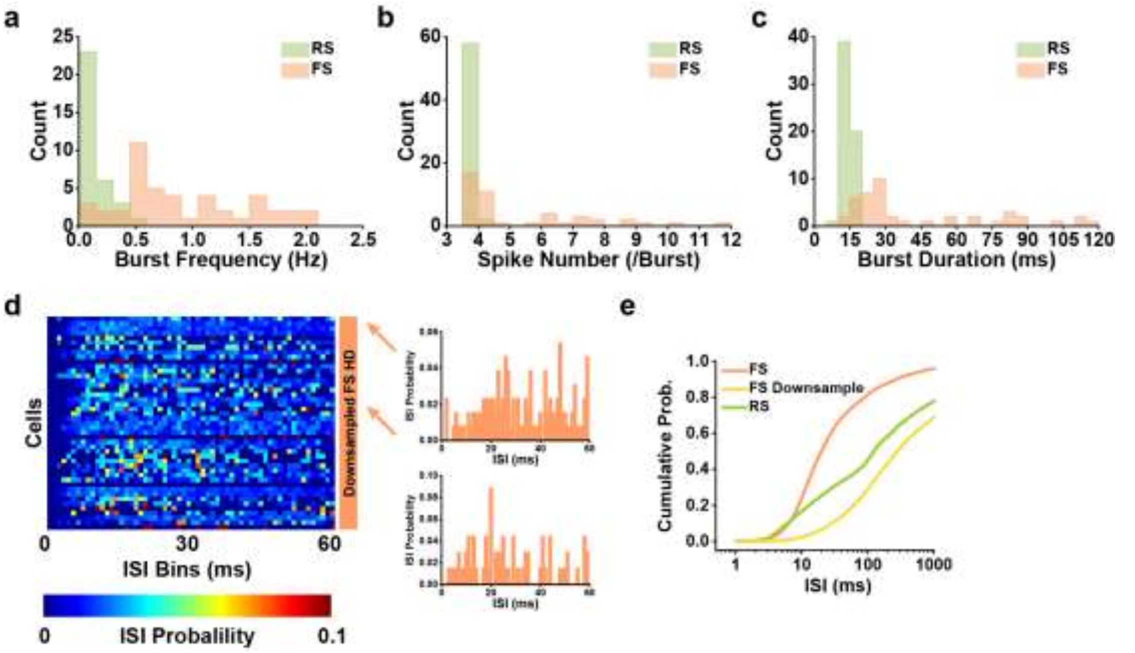

Figure S16. Downsampling FS HD cells disrupts bursty firing mode. a-c) Histograms showing the distributions of frequency of burst events (a), mean spike number in each burst (b), and mean burst duration (c) for both RS and FS HD cells. Continuously emitted spikes with interspike intervals (ISIs) within 20 ms are defined as burst events. d) ISIs histograms (left) of downsampled FS HD cells. Representative ISIs histograms (right) of two downsampled somatosensory FS HD cells (orange). e) Cumulative probability distribution of ISIs for FS, RS and downsampled FS HD cells.

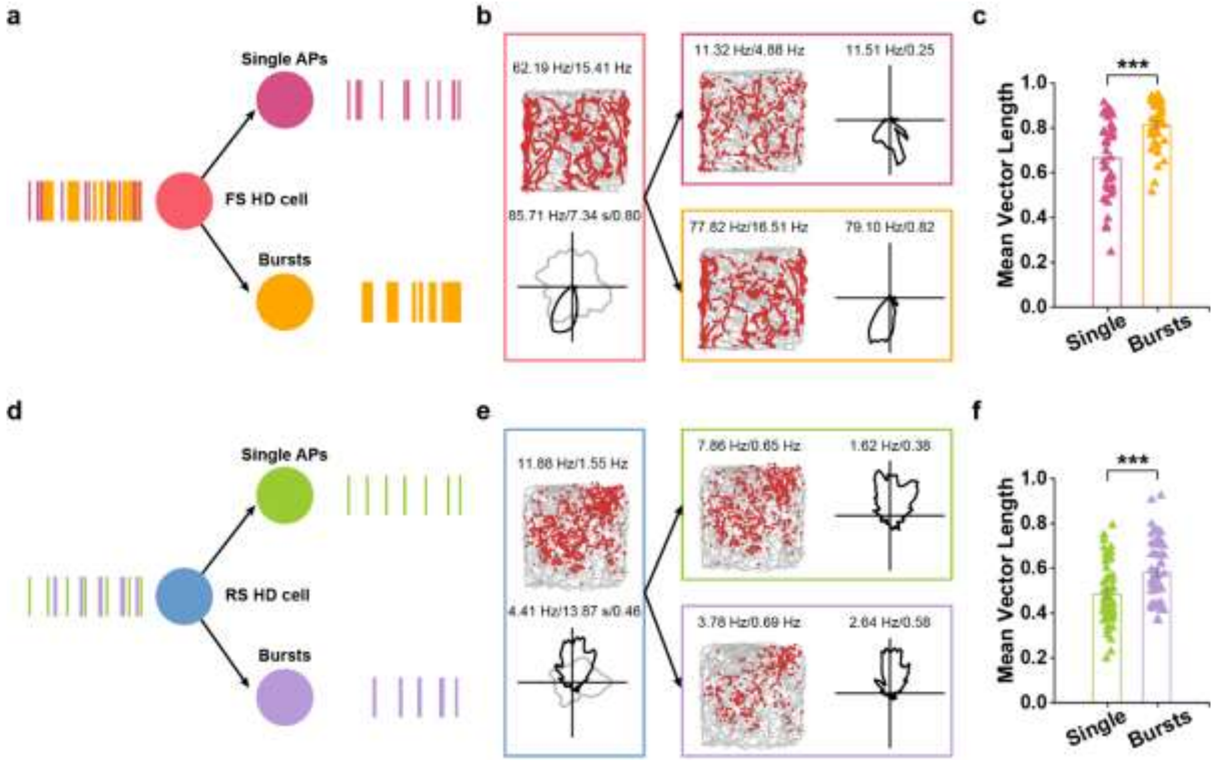

Figure S17. Bursts exhibit superior head direction tuning compared to single action potentials. a) Schematic showing the separation of a spike train into single action potentials (APs) and bursts. b) Head directional tuning of single APs (magenta) and bursts (orange) separated from the whole spike train (red) of a representative FS HD cell. Trajectory (grey line) with superimposed spike locations (red dots), and HD tuning curve (black) plotted against dwell-time (grey) are presented. Peak firing rate (fr), mean fr, peak angular fr, peak dwell time and mean vector length for each representative HD cell are labeled at the top of the panels. c) Higher mean vector length of bursts compared to single APs for FS HD cells. Data are shown in mean ± s.e.m. d-f) Same as (a-c) except for RS HD cells. Wilcoxon's signed-rank test, n = 45 and 60, respectively, $***P < 0.001$.

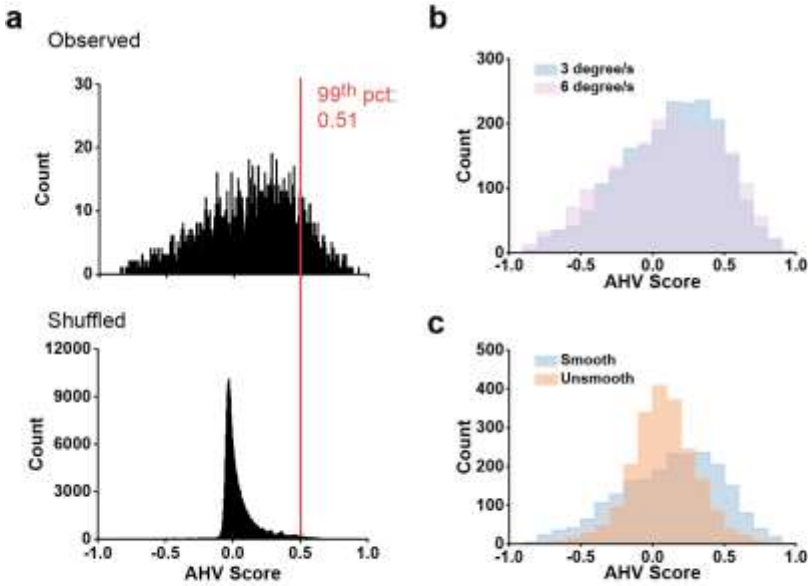

Figure S18. Classification of AHV cells and effect of smoothing and binning on AHV scores. a) Distribution of angular head velocity (AHV) scores for the entire pool of recorded S1 cells. The top panel shows the distribution for observed values. The bottom panel shows the distribution for randomly shuffled data from the same population. The red line and AHV score indicate 99th percentile for the shuffled data. Cutoff at 99th percentile was used as the threshold for the classification of AHV cells. b) Histograms showing the distributions of AHV scores for the classified AHV cells passing the threshold in (a) using bin sizes of 3 degree/s (blue) and 6 degree/s (purple) with smoothing. c) Histograms showing the distributions of AHV scores for the same AHV cells in (b) using bin size of 3 degree/s with (blue) and without (orange) smoothing. Note that the blue histograms in (b) and (c) are the same.

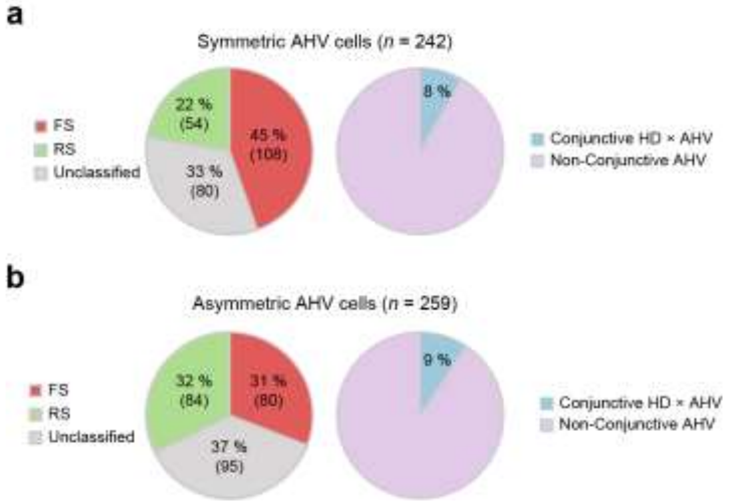

Figure S19. Classification of S1 AHV cells. a) Left, pie charts showing the proportions of FS, RS and unclassified cells of symmetric AHV cells. Right, conjunctive HD coding and non-conjunctive coding of AHV cells. b) Same as (a) for asymmetric AHV cells.

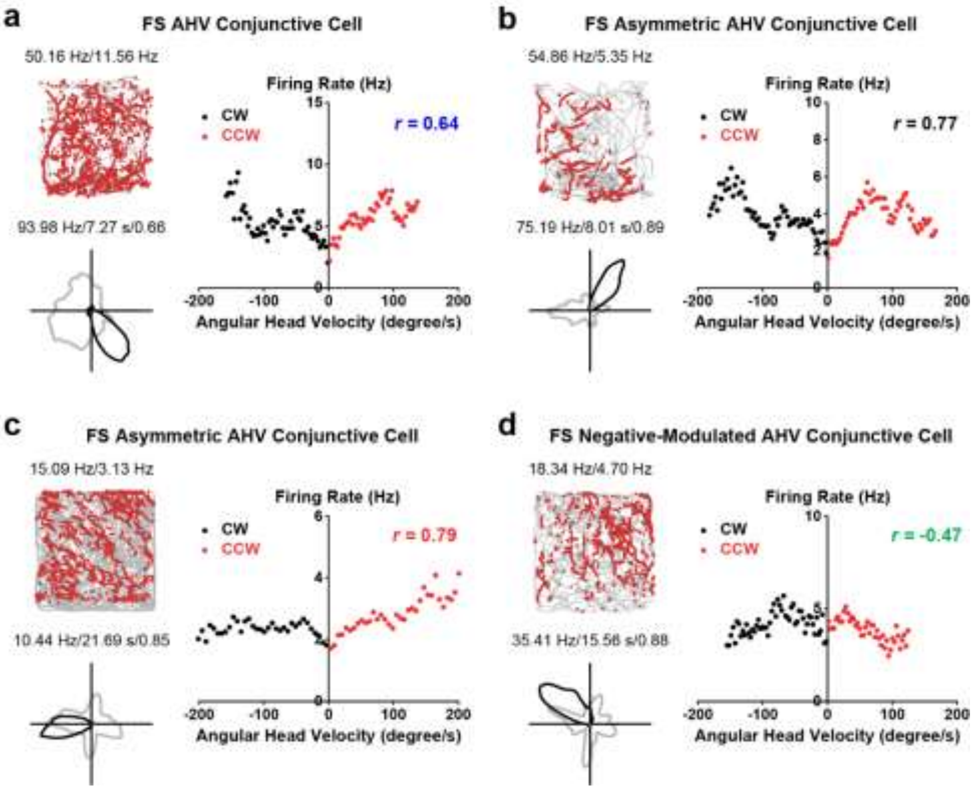

Figure S20. Conjunctive FS HD × AHV cells. a-d) Four representatives of conjunctive FS HD × AHV cells. a) FS HD cell with positive AHV modulation; b), FS HD cell with asymmetrical AHV modulation; c) FS HD cell with asymmetrical AHV modulation; d) FS HD cell with symmetrical negative AHV modulation. Trajectory (grey line) with superimposed spike locations (red dots; left top), and HD tuning curve (black) plotted against

dwell-time (grey; left bottom). Peak firing rate (fr), mean fr, peak angular fr, peak dwell time and mean vector length for each representative HD × AHV cell are labeled at the top of the panels. Right panel: Scatter plot of the firing rate versus the angular velocity of the representative cells.

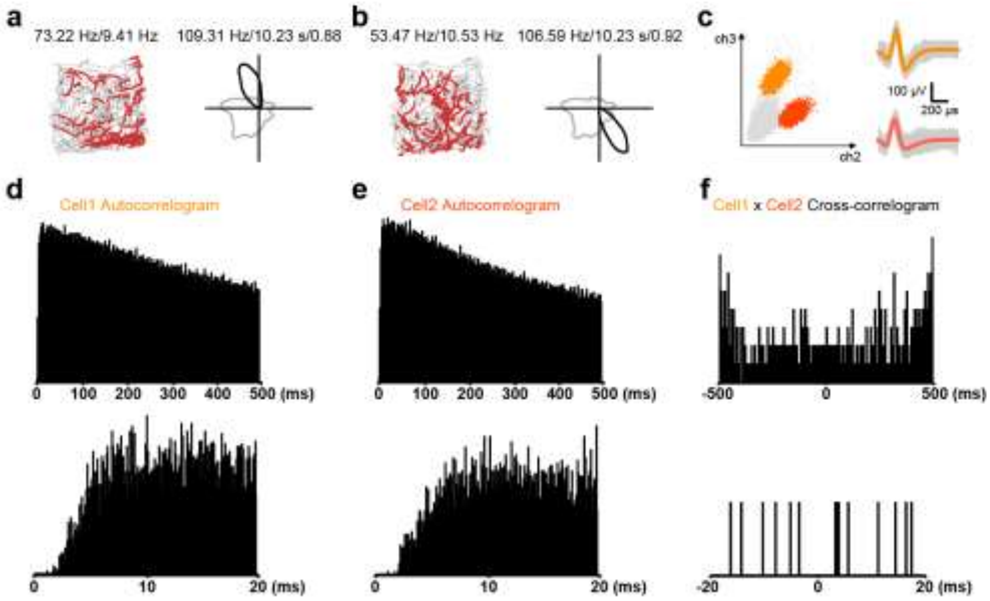

Figure S21. Two simultaneously recorded FS HD cells on the same tetrode. a, b) Simultaneously recorded FS HD cells from the same tetrode. Trajectory (grey line) with superimposed spike locations (red dots; left panel); and head direction tuning curves (black) plotted against dwell-time polar plot (grey; right panel). Peak firing rate (fr), mean fr, peak angular fr, peak dwell time and mean vector length for each representative HD cell are labeled at the top of the panels. c) Cluster diagrams and waveforms for the same cells. d, e) Representative spike-time autocorrelogram plots of two representative somatosensory FS HD cells. f), Representative spike-time cross-correlogram plots of two exampled cells above. Bottom panels of (d-f), shorter time scale of autocorrelogram and cross-correlogram.

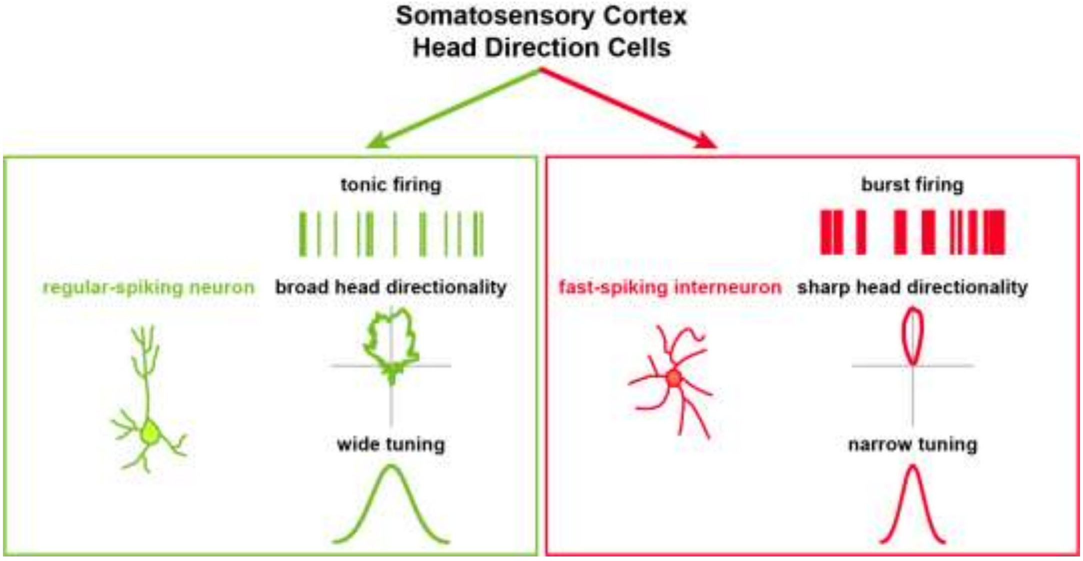

Figure S22. Fast-spiking interneurons exhibit superior head direction tuning compared to regular-spiking neurons. Both RS and FS HD cells are identified within the somatosensory cortex. The sharply tuned somatosensory HD cells are encoded by FS interneurons firing in a bursty mode (right) while those broadly tuned somatosensory HD cells are encoded by regular-spiking neurons firing in a tonic mode (left).
